# Superabsorption in an organic microcavity: Toward a quantum battery

**DOI:** 10.1126/sciadv.abk3160

**Published:** 2022-01-14

**Authors:** James Q. Quach, Kirsty E. McGhee, Lucia Ganzer, Dominic M. Rouse, Brendon W. Lovett, Erik M. Gauger, Jonathan Keeling, Giulio Cerullo, David G. Lidzey, Tersilla Virgili

**Affiliations:** 1 Institute for Photonics and Advanced Sensing and School of Chemistry and Physics, The University of Adelaide, South Australia 5005, Australia.; 2 Department of Physics and Astronomy, University of Sheffield, Hicks Building, Hounsfield Road, Sheffield S3 7RH, UK.; 3 Istituto di Fotonica e Nanotecnologia–CNR, IFN–Dipartimento di Fisica, Politecnico di Milano, Piazza Leonardo da Vinci 32, 20133 Milano, Italy.; 4 SUPA, School of Physics and Astronomy, University of St Andrews, St Andrews KY16 9SS, UK.; 5 SUPA, Institute of Photonics and Quantum Sciences, Heriot-Watt University, Edinburgh EH14 4AS, UK.

## Abstract

The rate at which matter emits or absorbs light can be modified by its environment, as markedly exemplified by the widely studied phenomenon of superradiance. The reverse process, superabsorption, is harder to demonstrate because of the challenges of probing ultrafast processes and has only been seen for small numbers of atoms. Its central idea—superextensive scaling of absorption, meaning larger systems absorb faster—is also the key idea underpinning quantum batteries. Here, we implement experimentally a paradigmatic model of a quantum battery, constructed of a microcavity enclosing a molecular dye. Ultrafast optical spectroscopy allows us to observe charging dynamics at femtosecond resolution to demonstrate superextensive charging rates and storage capacity, in agreement with our theoretical modeling. We find that decoherence plays an important role in stabilizing energy storage. Our work opens future opportunities for harnessing collective effects in light-matter coupling for nanoscale energy capture, storage, and transport technologies.

## Teaser

In a major step toward the development of a quantum battery, superabsorption has been achieved in an organic microcavity.

## INTRODUCTION

The properties of physical systems can typically be categorized as intensive (i.e., they are independent of the system size, such as density) or extensive (i.e., they grow in proportion to system size, such as mass). However, in some cases, cooperative effects can lead to superextensive scaling. A well-studied example of this is superradiant emission (*1*). In its original form, this describes emission from an ensemble of *N* emitters into free space. Constructive interference in the emission process means that the time for emission scales as 1/*N*, so that peak emission power is superextensive, scaling as *N*^2^. This behavior has been demonstrated on a number of platforms [low-pressure gases (*2*, *3*), quantum wells (*4*, *5*) and dots (*6*), J aggregates (*7*), Bose-Einstein condensates (*8*), trapped atoms (*9*), and nitrogen-vacancy centers (*10*)]. A less-studied example is superabsorption (*11*), describing the *N*-dependent enhancement of absorption of radiation by an ensemble of *N* two-level systems (TLSs). Only very recently has this been demonstrated for a small number of atoms (*12*). In principle, superabsorption could have important implications for energy storage and capture technologies, particularly if realized in platforms compatible with energy harvesting, such as organic photovoltaic devices. However, there are challenges in engineering the precise environment in which such behavior can occur and in monitoring the ultrashort charging time scales. Here, we show how these can be overcome, by combining organic microcavity fabrication with ultrafast pump-probe spectroscopy.

Superextensive scaling of energy absorption is also a key property of quantum batteries (QBs). These represent a new class of energy storage devices that operate on distinctly quantum mechanical principles. In particular, they are driven either by quantum entanglement, which reduces the number of traversed states in the Hilbert space compared to (classical) separable states alone (*13*–*21*), or by cooperative behavior that increases the effective quantum coupling between battery and source (*22*–*24*). These effects mean that QBs exhibit a charging time that is inversely related to the battery capacity. This leads to the intriguing idea that the charging power of QBs is superextensive, that is, it increases faster than the size of the battery. For a QB consisting of a collection of *N* identical quantum systems, a superextensive charging rate density (charging rate per subsystem) that scales as *N* or $\sqrt{N}$ in the thermodynamic limit (*20*) has been predicted.

Here, we experimentally realize a paradigmatic model proposed as a Dicke QB (*24*), which displays superextensive scaling of energy absorption, using an organic semiconductor as an ensemble of TLSs coupled to a confined optical mode in a microcavity. We also demonstrate how dissipation plays a crucial role; in a closed system, the coherent effects that lead to fast charging can also lead to subsequent fast discharging. Hence, stabilization of stored energy remains an open question: Proposed stabilization methods include continuous measurements (*25*), dark states (*21*), and novel energy trapping mechanisms (*26*, *27*). In our open noisy system, dephasing causes transitions between the optically active bright mode and inactive dark modes. This suppresses emission into the cavity mode, so that we have fast absorption of energy but slow decay, allowing retention of the stored energy until it can be used.

## RESULTS

### Device structure

**Fig. 1.**
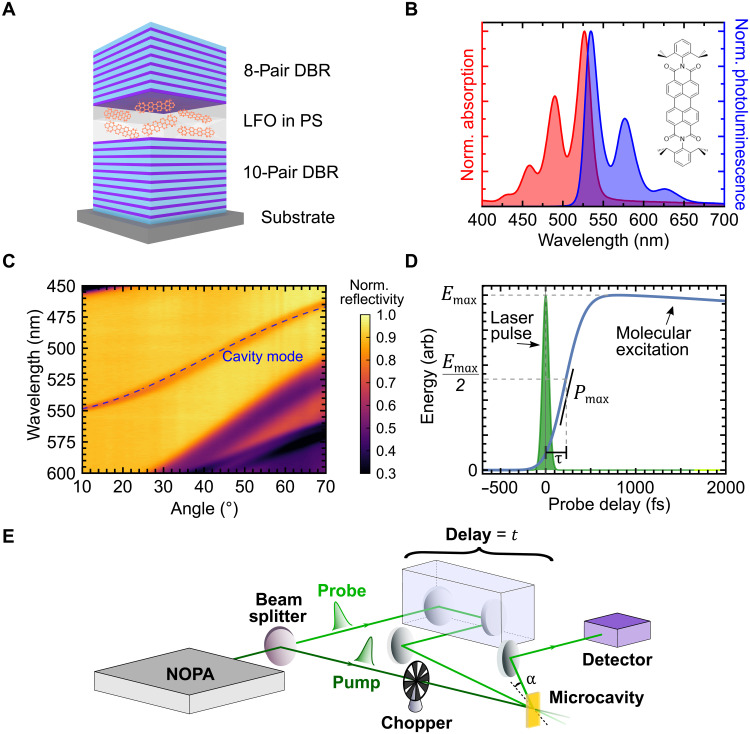
Schematics of the LFO microcavity and experimental setup. (**A**) Microcavity consisting of Lumogen-F orange (LFO) dispersed in a polystyrene (PS) matrix between distributed Bragg reflectors (DBRs). (**B**) Normalized absorption (red) and photoluminescence (blue) spectra for 1% concentration LFO film, with the molecular structure shown in the inset. We operate near peak absorption/photoluminescence. (**C**) Angle-dependent reflectivity of the 1% cavity, with a fit for the cavity mode shown by the blue dashed line. (**D**) A laser pump pulse excites the LFO molecules. The energetics of the molecules are then measured with probe pulses delayed by time *t*, from which we can ascertain the peak energy density (*E*_max_), rise time (τ), and peak charging power (*P*_max_). (**E**) Experimental setup for ultrafast transient reflectivity measurements. The output of a noncollinear optical parametric amplifier (NOPA) is split to generate pump (dark green) and probe (light green) pulses. A mechanical chopper is used to modulate the pump pulse to produce alternating pump-probe and probe-only pulses.

The structures fabricated consist of a thin (active) layer of a low-mass molecular semiconductor dispersed into a polymer matrix that is deposited by spin coating and positioned between two dielectric mirrors, forming a microcavity as illustrated schematically in Fig. 1A (see Materials and Methods for fabrication details). Organic semiconductors are particularly promising for many applications as the high oscillator strength and binding energy of molecular excitons mean that light can be absorbed efficiently, and excitons can exist at room temperature (*28*). The organic semiconductor used in this study was the dye Lumogen-F orange (LFO), whose chemical structure is shown in Fig. 1B. The normalized absorption and photoluminescence spectra for LFO dispersed at 1% concentration by mass in a polystyrene (PS) matrix are shown in Fig. 1B. By diluting the LFO, we reduce intermolecular interactions that lead to emission quenching, producing a high photoluminescence quantum yield of around 60% at low concentration (see fig. S1). The absorption peak at 526 nm and the emission peak at 534 nm correspond to the 0-0 transition, i.e., an electronic transition from and to the lowest vibrational state. Operating around the 0-0 transition, the LFO molecules can reasonably be considered as a TLS. We prepared samples with 0.5, 1, 5, and 10% concentrations, as these are representative of the optimal operating regimes; further increases in concentration lead to quenching, and signals from lower concentrations are indiscernible from noise. The absorption and photoluminescence spectra for the 0.5, 5, and 10% concentrations are given in fig. S2.

The optical microcavities fabricated support cavity modes whose energy is determined by the optical thickness of the LFO layer and the penetration of the optical field into the cavity mirrors (*29*). The confined photon field drives coherent interactions with the molecules, which underpin the collective effects that drive superabsorption. The LFO concentration dictates the operating coupling regime, with the 0.5 and 1% LFO cavities operating in the weak coupling regime, the 5% in the intermediate coupling regime, and the 10% in the strong coupling regime (see fig. S2 and discussion in Materials and Methods).

### Experimental setup

Charging and energy storage dynamics were measured using ultrafast transient-absorption spectroscopy (*30*), allowing femtosecond charging times to be measured. In this technique, we excite the microcavity with a pump pulse and then measure the evolution of stored energy (i.e., corresponding to the number of excited molecules) with a second probe pulse, delayed by time *t* (Fig. 1D). The probe pulse is transmitted through the top distributed Bragg reflector (DBR) of the cavity, and the reflection from the bottom DBR is measured. The differential reflectivity induced by the pump pulse is given by

$$\frac{\Delta R}{R}(t)=\frac{R_{\text{ON}}(t)-R_{\text{OFF}}}{R_{\text{OFF}}}$$ (1)

where *R*_ON_ (*R*_OFF_) is the probe reflectivity with (without) the pump excitation. Note that control films (active layers without the microcavities) are measured under differential transmittivity Δ*T* ∕ *T*. The control films will allow us to identify the underlying photophysics of the molecules.

In our experimental setup (shown schematically in Fig. 1E), transient-absorption measurements were performed in a degenerate, almost collinear configuration. Pump and probe pulses were generated by a broadband noncollinear optical parametric amplifier (NOPA) (*31*) and spanned the wavelength range of 500 to 620 nm with a nearly transform-limited sub–20-fs duration (further details in Materials and Methods). An optical delay line was used to control the probe delay time, and a mechanical chopper was used to modulate the pump pulse, providing alternating probe-only and pump-probe pulses, allowing us to measure pump-induced absorption changes. Measurements at different molecular concentrations were performed, adjusting the pump fluence to maintain an approximately constant photon density (i.e., pump photons per LFO molecule) *r* = *kN*_γ_/*N*, where *N* is the total number of molecules in the excitation volume, *N*_γ_ is the total number of pump laser photons, and *k* is the fraction of them that actually reaches the active layer of the microcavity. We estimate from the reflectivity data that only 6 to 8% of the initial pump excitation enters the cavity. We conducted our experiment in air at room temperature.

### Results

**Fig. 2.**
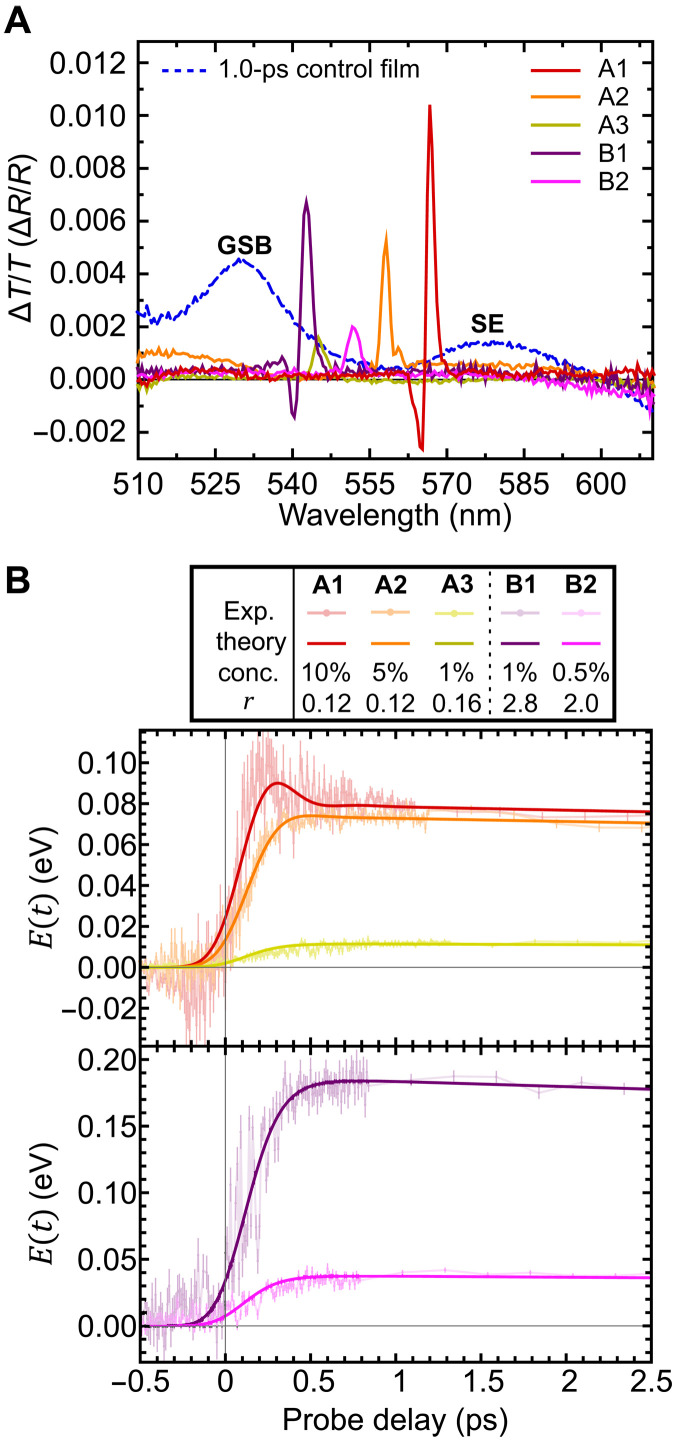
Experimental demonstration of superextensive charging. (**A**) Differential transmittivity (Δ*T*/*T*) spectra for the control film (at 1% LFO concentration) at a probe delay time of 1.0 ps and the differential reflectivity (Δ*R*/*R*) spectra for the microcavities at 1.25-ps probe delay. (**B**) Temporally resolved energy density of the microcavities shows that rise time decreases as stored energy density increases, indicating superextensive charging. A1, A2, and A3 label results for microcavities containing LFO at concentrations of 10, 5, and 1%, as the ratio of pump photons to molecules is kept approximately constant at *r* ≃ 0.14. B1 and B2 label measurements for LFO at concentrations of 1 and 0.5%, with *r* ≃ 2.4. The use of two different *r* values was necessary to achieve a sufficiently high signal-to-noise ratio. Points mark the experimental data, while continuous solid lines are the results of the theoretical model, with parameters given by a chi-squared minimization of the experimental data. Experimental uncertainties are estimated from the point-to-point variance of the data. GSB, ground state bleaching; SE, stimulated emission.

We first show that ultrafast transient-absorption spectroscopy can monitor the population of excited molecules, even in a cavity, by comparing the control film and the microcavity spectra as shown in Fig. 2A. A representative control film Δ*T*/*T* spectrum is shown for a probe delay time of 1.0 ps, and the Δ*R*/*R* spectra of the microcavities are shown at a delay of 1.25 ps (further data are given in the Supplementary Materials). We found the control film spectra at all concentrations to show two positive bands around 530 and 577 nm, which both reflect excited-state populations. By comparison with the spectra in Fig. 1B, we attribute the 530-nm band to ground state bleaching, i.e., suppression of absorption due to molecules already being in their excited state. The 577-nm band instead corresponds to stimulated emission by excited molecules. For each of the microcavity spectra, we have a single prominent peak, which corresponds to the transient signal filtered by the cavity mode. This implies that the time-dependent transient reflectivity signal is proportional to the change in the number of excited molecules created by the pump (*32*), i.e., $\frac{\Delta R}{R}(t)\propto N_{↑}(t)$. Since the energy stored in the molecules is also proportional to the number of excited molecules *E*(*t*) ∝ *N*_↑_(*t*), we can thus monitor the stored energy. While the experiment directly provides the time dependence, estimating the absolute scale of energy density requires multiplying Δ*R*/*R* by a time-independent constant. Estimating this constant from first principles is challenging, so we instead extract it through fitting to the theoretical model, which is discussed below. This fitting is discussed in section S3. We also note that two of the microcavity spectra show a negative Δ*R*/*R* band, which results from the change in the refractive index induced by the pump pulse (*33*).

Figure 2B shows the experimental values for the time-dependent stored energy density. In all microcavities studied, the energy density undergoes a rapid rise followed by slow decay. The time scale of the rapid rise varies with concentration. We adjust the laser power to fix photon density *r* across comparable microcavities and compare behavior with different LFO concentrations. Details of how *r* is estimated are provided in the Supplementary Materials. We found that to achieve a sufficiently high signal-to-noise ratio, it was not possible to compare all microcavities at the same *r* value; instead, a constant *r* value was maintained for matched structures. Specifically, measurements were made on microcavities with LFO concentrations of 10, 5, and 1% with approximately constant *r* ≃ 0.14 (respectively labeled as A1, A2, and A3), and 1 and 0.5% with *r* ≃ 2.4 (labeled as B1 and B2).

Overlaying the experimental data are the corresponding theoretical predictions (see the “Theoretical model” section). To account for both the instrument response time (~20 fs) and the cavity photon lifetime (which was estimated as discussed in the Supplementary Materials), the theoretical curves are convolved with a Gaussian response function with a full width at half maximum of ~120 fs. There is good agreement between the experimental data and the corresponding theoretical curves.

To obtain energetic dynamics, we take away the response function from the theoretical fit, as shown in fig. S15. Table 1 summarizes the rise time or the time to reach half maximum energy (τ), the peak stored energy density (*E*_max_), and the charging rate or peak charging power density [*P*_max_ = max (*dE* ∕ *dt*)]. These are extracted from the theoretical fit to the data presented in Fig. 2B. We see that τ decreases with *N*, while *E*_max_ and *P*_max_ increase with *N*. Recalling that *E*_max_ and *P*_max_ are the stored energy and charging power per molecule, this indicates superextensive behavior. The scaling with *N* is not the same across all experiments, and in table S2, we summarize the different scaling.

**Table 1.** Summary of the experimental results. In each experimental groupings A,B the number of molecules (*N*) increases while the ratio of photons to molecules remains constant (*r* ≈ 0.105 and 2.4, respectively). The rise time τ is defined by the time to reach *E*_max_/2, where *E*_max_ is the peak stored energy per molecule or energy density. The charging rate *P*_max_ = max(*dE*/*dt*) is the peak charging power per molecule or charging power density.

**Exp.**	***N*(×10^10^)**	**τ (ps)**	***E*_max_ (eV**)	***P*_max_ (eV/ps)**
**A1**	16.0	0.094	0.108	0.791
**A2**	8.1	0.120	0.076	0.412
**A3**	1.6	0.118	0.011	0.060
**B1**	0.16	0.114	0.184	1.008
**B2**	0.21	0.105	0.037	0.221

Our results demonstrate that as the number of molecules in the microcavity increases, its charging power density remarkably increases. This means that it takes less time to charge a single microcavity containing *N* molecules than it would to charge *N* single-molecule microcavities, even if the latter were charged simultaneously. Furthermore, one microcavity with *N* molecules would store more energy than *N* microcavities, where each contained a single molecule. These superextensive properties are the key experimental findings of our work and are supported by the theoretical modeling presented in the next section.

### Theoretical model

The experimental dynamics can be reproduced by modeling, with the Lindblad master equation, the *N* TLSs in an optical cavity with light-matter coupling strength *g*, a driving laser with a Gaussian pulse envelope and peak amplitude η_0_, and three decay channels corresponding to the cavity decay (κ), TLS dephasing (γ*^z^*), and TLS relaxation (γ^−^). To solve this many-body Lindblad master equation, we make use of the cumulant expansion (*34*–*36*), with model parameters given by a chi-squared minimization of the experimental data. Experimental uncertainties are estimated from the point-to-point variance of the data. Further details can be found in Materials and Methods and in the Supplementary Materials.

**Fig. 3.**
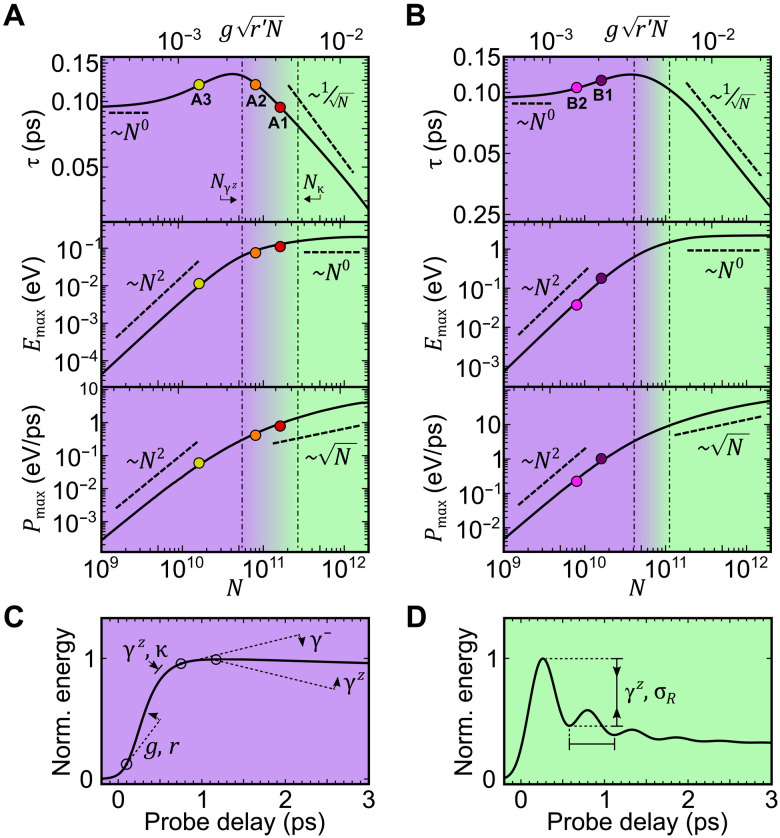
Charging dynamics as a function of the number of molecules. (**A** and **B**) Theoretical model (solid line) for *r* = 0.14 and 2.4, respectively. We show three operating regimes: decay-dominated (purple), coupling-dominated (green), and a decay-coupling-crossover regime. The decay-dominated regime is bounded by *N*_κ_ < κ^2^/*g*^2^*r*′, and the coupling-dominated regime is bounded by *N*_γ*^z^*_ > γ^*z*2^/*g*^2^*r*′, where *r* ′ = max (1, *r*). The colored dots indicate where the experiments sit on these curves. The uncertainty in *N* is 10%, which is smaller than the dot size. (**C**) qualitatively depicts the effects of the model parameters in shaping the dynamics in the decay-dominated regime. (**D**) qualitatively depicts the effects of the additional model parameters in shaping the dynamics in the coupling-dominated regimes. σ_R_ is the temporal width of the instrument response function.

From our cumulant expansion simulations, we show how τ, *E*_max_, and *P*_max_ vary as a function of *N* in Fig. 3 (A and B). The interplay among the decay channels, driving laser, and cavity couplings gives rise to a rich set of behaviors. We identify three regimes: decay-dominated at small *N* and coupling-dominated at large *N*, along with a crossover regime between them. The system exhibits superextensive energy density scaling in the decay-dominated regime and subextensive charging time in the coupling-dominated regime. In the crossover regime, the system exhibits both superextensive energy density scaling and subextensive charging times. Charging power density is superextensive in all regimes.

Figure 3 (C and D) shows the typical time dependence in decay-dominated and coupling-dominated regimes, indicating how the model parameters affect the dynamics. In particular, the presence of the decay channels gives rise to ratchet states that are capable of absorbing but not emitting light (*37*), thereby allowing the energy to be stably stored. See Materials and Methods and the Supplementary Materials for further discussion on the operating regimes. Figure 3 is augmented with an animation of how the energetic dynamics changes with *N* (see movie S1).

Figure 3 (A and B) provides an explanation for the different scaling of the observables with *N* in Table 1. Specifically, A1 and A2 operate in the coupling-dominated regime, where τ scales slightly less than *N*^−1/2^, *E*_max_ scales slightly more than *N*^0^, and *P*_max_ scales slightly more than *N*^1/2^. For the region between A2 and A3, the average scaling of τ falls between *N*^0^ and *N*^−1/2^, *E*_max_ between *N*^2^ and *N*^0^, and *P*_max_ between *N*^2^ and *N*^1/2^. As A2 is further into the coupling-dominated regime than A3 is into the decay-dominated regime, the average scaling values between A2 and A3 are skewed toward the coupling-dominated scalings. B1 and B2 operate in the crossover regime, with an average scaling with *N* that is between the decay-dominated and coupling-dominated scalings, as reflected in Table 1.

### Discussion

We have provided direct experimental evidence of superextensive energy storage capacity and charging in an organic microcavity by using ultrafast optical spectroscopy. Our realization of a prototype Dicke QB highlights the fact that purely closed unitary dynamics is insufficient for realizing a practical QB. The retention of energy requires finely tuned decoherence processes, allowing the battery to charge quickly and yet discharge much more slowly. This stabilization of stored energy is a key step to exploit superextensive charging. Our observation of dephasing shows that realistic noisy environments can aid the implementation and application of useful QBs. A challenge for future work is to explore further how concepts of ratchet states could keep a QB operating in the range of higher-lying energy states that are associated with maximum absorption enhancement, i.e., near the midpoint of the Dicke ladder (*37*).

We conclude by discussing the potential for future applications based on superextensive charging. One practical challenge noted above is that quenching limits the performance of the QB at high concentrations. Overcoming this limitation requires careful choice of materials to suppress intermolecular quenching. We note that there are classes of materials where quenching is particularly suppressed. For example, in proteins such as green fluorescent protein (*38*), the active chromophore is surrounded by a cage, which suppresses exciton-exciton quenching at high intensities. These materials might provide a route to allow the study of higher concentrations. Beyond energy storage, the key challenge for practical applications of this effect is its integration in devices where energy can be efficiently extracted and used. While our focus has been on the quantum advantage in charging, there do exist approaches to efficiently extract energy. For example, this may be achieved by including charge transport layers between the active layer and the cavity layers (*39*). The transport layers allow charge separation of the excitons as well as preventing recombination. This transforms the top cavity layer into a cathode and the bottom cavity into an anode, giving rise to an electric current. Hence, our work provides a direct path for the integration of the superextensive energy absorption process in an organic photovoltaic device. The fast dynamics of such a device may also be useful as an optical sensor in low-light conditions or potentially for energy harvesting applications (*40*–*43*). More generally, the idea of superextensive charging may have wide-reaching consequences for sensing and energy capture and storage technologies.

## MATERIALS AND METHODS

### Device fabrication

The microcavities constructed consist of a thin layer of LFO (Kremer Pigmente) dispersed in a PS (Sigma-Aldrich; average molecular weight of ~192,000) matrix. The bottom DBR consisted of 10 pairs of SiO_2_/Nb_2_O_5_ and were fabricated using a mixture of thermal evaporation and ion-assisted electron beam deposition by Helia Photonics Ltd. Solutions of LFO dissolved in PS (25 mg/ml) in dichloromethane were prepared at 0.5, 1, 5, and 10% concentration by mass. Each LFO solution was then spin coated on top of the bottom DBR to produce a thin film with an approximate thickness of 185 nm. An eight-pair DBR was then deposited on top of the LFO layer using electron beam deposition. With this pair of mirrors, the reflectivity was >99% in the spectral region of interest (*44*).

The diluted molecules are expected to be isolated at a low concentration of 0.1 to 1%, but at higher dye concentrations, the 0-0 emission transition red-shifts by a few nanometers, and the second peak increases in intensity because of aggregation of the dye molecules. This is evident in fig. S2 (A and B), with additional broader features observed at longer wavelengths, which we assign to intermolecular states such as excimers.

The 0.5 and 1% cavities lie in the weak coupling regime, i.e., no polaritonic splitting could be seen in the cavity reflectivity spectrum, as shown in fig. S2. For the 5% cavity, we see a weak anticrossing feature in the reflectivity spectrum (a small kink near the crossing), indicating operation in the intermediate coupling regime. The 10% cavity operated in the strong coupling regime, showing a Rabi splitting of around 100 meV around the 0-0 transition (along with intermediate coupling between the cavity mode and the 0-1 transition). Figure S3 shows a transfer matrix simulation of the electric field distribution of the 1% cavity (the cavities exhibit similar distributions).

### Pump-probe spectroscopy

Probe and pump pulses were generated by a NOPA. The NOPA was pumped by a fraction (450 μJ) of the laser beam generated by a regeneratively amplified Ti:Sapphire laser (Coherent Libra) producing 100-fs pulses at 800 nm at a repetition rate of 1 kHz. A pair of chirped mirrors were placed at the output of the NOPA to compensate for temporal dispersion, and by using seven “bounces,” we were able to generate pulses with a temporal width below 20 fs. The laser beam was then split by a beam splitter, with the probe being delayed via a translation stage and the pump being modulated mechanically using a chopper at 500 Hz.

### Lindblad master equation

As noted above, we find that the experimental behavior is well reproduced by the dynamics of the Dicke model, a model of a microcavity photon mode coupled to TLSs representing the molecules. As further discussed in the Supplementary Materials, such a model is generally an approximation for organic molecules but for some systems can become a very accurate approximation in the limit of low temperatures (*45*).

The open driven nature of the experimental system is modeled with the Lindblad master equation

$$\overset{\cdot }{\rho }(t)=-\frac{i}{\hbar }[H(t),\rho (t)]+\sum_{j=1}^{N}(\gamma^{z}L[\sigma_{j}^{z}]+\gamma^{-}L[\sigma_{j}^{-}])+\kappa L[a]$$ (2)

where ρ(*t*) is the density matrix, and $L[O]\equiv O\rho \;O^{†}-\frac{1}{2}O^{†}O\rho -\frac{1}{2}\rho \;O^{†}O$ is the Lindbladian superoperator. *a*^†^ and *a* are the cavity photon creation and annihilation operators, and $\sigma_{j}^{x,y,z}$are the Pauli spin matrices for each molecule, with the raising and lowering spin operators defined as $\sigma_{j}^{\pm }=(\sigma_{j}^{x}\pm i\sigma_{j}^{y})/2$. There are three decay channels corresponding to the cavity decay (κ), dephasing (γ*^z^*), and relaxation rate (γ^−^) of the individual TLSs. The Hamiltonian for the LFO molecules in cavity is modeled as a collection of noninteracting TLSs with characteristic frequency ω equal to that of the cavity mode and resonantly coupled to the cavity with strength *g*. The molecules are driven by a laser described by a Gaussian pulse envelope $\eta (t)=\frac{\eta_{0}}{\sigma \sqrt{2\pi }}e^{-\frac{1}{2}{\left(\frac{t-t_{0}}{\sigma }\right)}^{2}}$ and a carrier frequency ω*_L_*. We work in the frame of the laser carrier frequency, and so write

$$H(t)=\hbar \Delta a^{†}a+\sum_{j=1}^{N}[\frac{\hbar \Delta }{2}\sigma_{j}^{z}+g(a^{†}\sigma_{j}^{-}+a\sigma_{j}^{+})]+i\hbar \eta (t)(a^{†}-a)$$ (3)

where Δ = ω − ω*_L_* is the detuning of the cavity frequency from the laser driving frequency. The LFO molecules are initially in the ground state, and the laser is on resonance (Δ = 0).

### Cumulant expansion

The energy density of the cavity containing identical molecules with transition energy ω is $E(t)=\frac{\hbar \omega }{2}[\langle \sigma^{z}(t)\rangle +1]$. In general, the equation of motion $\left(\partial /\partial t)\langle \sigma^{z}\rangle =\mathrm{Tr}[\sigma^{z}\overset{\cdot }{\rho }\right]$ depends on both the first-order moments ⟨σ^*x*, *y*, *z*^⟩ and ⟨*a*⟩ and higher-order moments, leading to a hierarchy of coupled equations. Within mean field theory, the second-order moments are factorized as ⟨*AB*⟩ = ⟨*A*⟩⟨*B*⟩, which closes the set of equations at first order. This approximation is valid at large *N*, as corrections scale as 1/*N*. To capture the leading order effects of finite sizes, we make a second-order cumulant expansion (*34*–*36*), i.e., we keep second-order cumulants ⟨⟨*AB*⟩⟩ = ⟨*AB*⟩ − ⟨*A*⟩⟨*B*⟩ and assume that the third-order cumulants vanish, which allows us to rewrite third-order moments into products of first- and second-order moments (*46*). In our experiments, the number of molecules in the cavity is large (>10^10^), and we find that higher-order correlations are negligible. We give the equations of motion up to second order in the Supplementary Materials.

### Operating regimes

The decay-dominated (purple region in Fig. 3, A and B) regime occurs when the collective light-matter coupling is weaker than the decay channels, $g\sqrt{\mathrm{Nr}\prime }<\{\kappa ,\gamma^{z},\gamma^{-}\}$, where *r* ′ = max (1, *r*). In this regime, the time scale of cavity dynamics is slow relative to the decay rate. Figure 3C shows a typical time dependence in this regime, indicating how the model parameters affect the dynamics. In this regime, the increase in the effective coupling relative to the decay strength sees an *N*^2^ superextensive scaling of the energy and power density, while rise time remains constant. Experiment A3 operates near the boundary of this regime (Fig. 3A).

In the coupling-dominated (green region in Fig. 3, A and B) regime, the effective collective light-matter coupling $g\sqrt{\mathrm{Nr}\prime }>\{\gamma^{z},\gamma^{-},\kappa \}$ dominates over the decay channels. In this regime, the time scale of cavity dynamics is fast relative to the decay rate, and we observe $\sqrt{N}$-superextensive power scaling and $1/\sqrt{N}$ dependence of rise time, while the maximum energy density remains constant. While power scaling is superextensive in both regimes, the origin of this differs: For the decay-dominated regime, this is the result of the superextensive energy scaling, while for the coupling-dominant regime, it is the result of a superextensive decrease in the rise time. Experiments A1 and A2 operate in this regime (Fig. 3A).

In the crossover between the regimes (purple-green), the collective coupling falls between the cavity decay rate and the TLS dephasing rate, $\{\kappa ,\gamma^{-}\}<g\sqrt{\mathrm{Nr}\prime }<\gamma^{z}$. In Fig. 3 (A and B), γ^−^ is small such that $g\sqrt{\mathrm{Nr}\prime }\gg \gamma^{-}$ for all values of *N*, and so, there is no boundary labeled for this decay rate. In this case, capacity and rise time can simultaneously scale super- and subextensively, but at a rate slower than in the decay and coupling-dominated regimes, respectively. Experiments B1 and B2 operate in this regime (Fig. 3B).

### Decay and coupling rates

The parameters needed in the theory calculations are the cavity leakage rate κ, the dephasing rate γ*^z^*, the nonradiative decay rate γ^−^, the interaction strength *g*, and the temporal width of the instrument response function σ_R_. Note that the temporal width of the pump pulse is fixed at σ = 20 fs. For the dephasing rate, we note that as one enters the strong coupling regime, exciton delocalization suppresses the effect of dephasing (*47*). To approximately capture this effect, we assume that the dephasing rate scales with the number of molecules as γz=γ0z(N5%N), where $\gamma_{0}^{z}$ is taken to be constant, and *N*_5%_ is the number of molecules in the 5% cavity. The experimental uncertainty in *N* is estimated to be 10%. The cavity lifetime *T* comes into the model in both σ_R_ = *T* and the cavity leakage rate κ = 1/*T*. From transfer matrix modeling on the 1 and 0.5% cavities (where polariton effects are small), we estimate that *T* ≈ 306 fs. However, on the basis of the measured finesse of the cavities, we estimate that *T* = 120 fs. Transfer matrix modeling assumes perfectly smooth mirrors, while measured finesse includes inhomogeneous broadening effects, neither of which we want to include in κ and σ_R_. In the following optimization, we therefore assume that *T* ∈ [120,306] fs, with lower values more likely due to transfer matrix calculations being prone to error.

For *T* values within this range, the remaining three parameters in the model ($\gamma_{0}^{z}$, γ^−^, and *g*) were found through a global chi-squared optimization, simultaneously optimizing over all experiments. Uncertainties in these fitting parameters were then estimated by using the reduced ${\tilde{\chi }}^{2}$ distribution to find the 68% confidence interval of the model parameters. This corresponds to the range ${\tilde{\chi }}^{2}\leq {\tilde{\chi }}_{\text{min}}^{2}+\text{∆}$, where for a three-parameter optimization and *k* total data points, ∆ ≈ 3.51/(*k* − 3) (*48*). In the Supplementary Materials, we present a figure showing the minimum reduced chi-squared value as a function of *T*, and for each point, we show the optimal set of parameters ($\gamma_{0}^{z}$, γ^−^, and *g*) along with the 68% confidence intervals. From this, and by comparison of the experimentally measured and theoretically calculated reflectivity for each parameter set, we concluded that the lifetime most representative of the data was *T* = 120 fs, with $\gamma^{-}=(0.0141_{-0.0024}^{+0.0031})$ meV, $g=(10.6_{-1.3}^{+2.2})$ neV, and $\;\gamma_{0}^{z}=(1.68_{-0.18}^{+0.25})$ meV. See the Supplementary Materials for more details.

## Supplementary Materials

### This PDF file includes:

Sections S1 to S4 Tables S1 to S3 Figs. S1 to S17 Legend for movie S1 References

## References

[1] M. Gross, S. Haroche, Superradiance: An essay on the theory of collective spontaneous emission. Phys. Rep. 93, 301–396 (1982).

[2] N. Skribanowitz, I. P. Herman, J. C. MacGillivray, M. S. Feld, Observation of Dicke superradiance in optically pumped HF Gas. Phys. Rev. Lett. 30, 309–312 (1973).

[3] H. M. Gibbs, Q. H. F. Vrehen, H. M. J. Hikspoors, Single-pulse superfluorescence in cesium. Phys. Rev. Lett. 39, 547–550 (1977).

[4] J. Feldmann, G. Peter, E. O. Gobel, P. Dawson, K. Moore, C. Foxon, R. J. Elliott, Linewidth dependence of radiative exciton lifetimes in quantum-wells. Phys. Rev. Lett. 59, 2337–2340 (1987). 10035517 10.1103/PhysRevLett.59.2337

[5] B. Deveaud, F. Clerot, N. Roy, K. Satzke, B. Sermage, D. S. Katzer, Enhanced radiative recombination of free excitons in GaAs quantum wells. Phys. Rev. Lett. 67, 2355–2358 (1991). 10044405 10.1103/PhysRevLett.67.2355

[6] T. Itoh, M. Furumiya, Size-dependent homogeneous broadening of confined excitons in cucl microcrystals. JOL 48-49, 704–708 (1991).

[7] S. Deboer, D. A. Wiersma, Dephasing-induced damping of superradiant emission in J-aggregates. Chem. Phys. Lett. 165, 45–53 (1990).

[8] S. Inouye, A. P. Chikkatur, D. M. Stamper-Kurn, J. Stenger, D. E. Pritchard, W. Ketterle, Superradiant Rayleigh scattering from a Bose-Einstein condensate. Science 285, 571–574 (1999). 10417384 10.1126/science.285.5427.571

[9] R. Reimann, W. Alt, T. Kampschulte, T. Macha, L. Ratschbacher, N. Thau, S. Yoon, D. Meschede, Cavity-modified collective Rayleigh scattering of two atoms. Phys. Rev. Lett. 114, 023601 (2015). 25635545 10.1103/PhysRevLett.114.023601

[10] A. Angerer, K. Streltsov, T. Astner, S. Putz, H. Sumiya, S. Onoda, J. Isoya, W. J. Munro, K. Nemoto, J. Schmiedmayer, J. Majer, Superradiant emission from colour centres in diamond. Nat. Phys. 14, 1168–1172 (2018).

[11] K. D. Higgins, S. C. Benjamin, T. M. Stace, G. J. Milburn, B. W. Lovett, E. M. Gauger, Superabsorption of light via quantum engineering. Nat. Commun. 5, 4705 (2014). 25146588 10.1038/ncomms5705 PMC4143938

[12] D. Yang, S.-h. Oh, J. Han, G. Son, J. Kim, J. Kim, M. Lee, K. An, Realization of superabsorption by time reversal of superradiance. Nat. Photonics 15, 272–276 (2021).

[13] R. Alicki, M. Fannes, Entanglement boost for extractable work from ensembles of quantum batteries. Phys. Rev. E Stat. Nonlin. Soft Matter Phys. 87, 042123 (2013). 23679388 10.1103/PhysRevE.87.042123

[14] K. V. Hovhannisyan, M. Perarnau-Llobet, M. Huber, A. Acin, Entanglement generation is not necessary for optimal work extraction. Phys. Rev. Lett. 111, 240401 (2013). 24483629 10.1103/PhysRevLett.111.240401

[15] F. C. Binder, S. Vinjanampathy, K. Modi, J. Goold, Quantacell: Powerful charging of quantum batteries. New J. Phys. 17, 075015 (2015).

[16] G. M. Andolina, D. Farina, A. Mari, V. Pellegrini, V. Giovannetti, M. Polini, Charger-mediated energy transfer in exactly solvable models for quantum batteries. Phys. Rev. B 98, 205423 (2018).

[17] G. M. Andolina, M. Keck, A. Mari, M. Campisi, V. Giovannetti, M. Polini, Extractable work, the role of correlations, and asymptotic freedom in quantum batteries. Phys. Rev. Lett. 122, 047702 (2019). 30768349 10.1103/PhysRevLett.122.047702

[18] R. Alicki, A quantum open system model of molecular battery charged by excitons. J. Chem. Phys. 150, 214110 (2019). 31176352 10.1063/1.5096772

[19] Y. Y. Zhang, T. R. Yang, L. Fu, X. Wang, Powerful harmonic charging in a quantum battery. Phys. Rev. E 99, 052106 (2019). 31212558 10.1103/PhysRevE.99.052106

[20] F. Campaioli, F. A. Pollock, F. C. Binder, L. Celeri, J. Goold, S. Vinjanampathy, K. Modi, Enhancing the charging power of quantum batteries. Phys. Rev. Lett. 118, 150601 (2017). 28452497 10.1103/PhysRevLett.118.150601

[21] J. Q. Quach, W. J. Munro, Using dark states to charge and stabilize open quantum batteries. Phys. Rev. Appl. 14, 024092 (2020).

[22] T. P. Le, J. Levinsen, K. Modi, M. M. Parish, F. A. Pollock, Spin-chain model of a many-body quantum battery. Phys. Rev. A 97, 022106 (2018).

[23] X. Zhang, M. Blaauboer, Enhanced energy transfer in a Dicke quantum battery. arXiv:1812.10139 (2018).

[24] D. Ferraro, M. Campisi, G. M. Andolina, V. Pellegrini, M. Polini, High-power collective charging of a solid-state quantum battery. Phys. Rev. Lett. 120, 117702 (2018). 29601745 10.1103/PhysRevLett.120.117702

[25] S. Gherardini, F. Campaioli, F. Caruso, F. C. Binder, Stabilizing open quantum batteries by sequential measurements. Phys. Rev. Res. 2, 013095 (2020).

[26] A. C. Santos, A. Saguia, M. S. Sarandy, Stable and charge-switchable quantum batteries. Phys. Rev. E 101, 062114 (2020). 32688466 10.1103/PhysRevE.101.062114

[27] W. M. Brown, E. M. Gauger, Light harvesting with guide-slide superabsorbing condensed-matter nanostructures. J. Phys. Chem. Lett. 10, 4323–4329 (2019). 31251067 10.1021/acs.jpclett.9b01349

[28] D. Sanvitto, S. Kéna-Cohen, The road towards polaritonic devices. Nat. Mater. 15, 1061–1073 (2016). 27429208 10.1038/nmat4668

[29] V. Savona, L. C. Andreani, P. Schwendimann, A. Quattropani, Quantum well excitons in semiconductor microcavities: Unified treatment of weak and strong coupling regimes. Solid State Commun. 93, 733–739 (1995).

[30] G. Cerullo, C. Manzoni, L. Lüer, D. Polli, Time-resolved methods in biophysics. 4. Broadband pump–probe spectroscopy system with sub-20 fs temporal resolution for the study of energy transfer processes in photosynthesis. Photochem. Photobiol. Sci. 6, 135–144 (2007). 17277836 10.1039/b606949e

[31] C. Manzoni, G. Cerullo, Design criteria for ultrafast optical parametric amplifiers. J. Opt. 18, 103501 (2016).

[32] O. Svelto, D. C. Hanna, *Principles of Lasers* (Springer, 2010), vol. 1.

[33] T. Virgili, D. G. Lidzey, D. D. C. Bradley, G. Cerullo, S. Stagira, S. De Silvestri, An ultrafast spectroscopy study of stimulated emission in poly(9,9-dioctylfluorene) films and microcavities. Appl. Phys. Lett. 74, 2767–2769 (1999).

[34] P. Kirton, J. Keeling, Suppressing and restoring the Dicke superradiance transition by dephasing and decay. Phys. Rev. Lett. 118, 123602 (2017). 28388206 10.1103/PhysRevLett.118.123602

[35] K. B. Arnardottir, A. J. Moilanen, A. Strashko, P. Törmä, J. Keeling, Multimode organic polariton lasing. arXiv:2004.06679 (2020). 10.1103/PhysRevLett.125.233603 33337197

[36] M. Zens, D. O. Krimer, S. Rotter, Critical phenomena and nonlinear dynamics in a spin ensemble strongly coupled to a cavity. II. Semiclassical-to-quantum boundary. Phys. Rev. A 100, 013856 (2019).

[37] K. D. B. Higgins, B. W. Lovett, E. M. Gauger, Quantum-enhanced capture of photons using optical ratchet states. J. Phys. Chem. C 121, 20714–20719 (2017).

[38] C. P. Dietrich, A. Steude, L. Tropf, M. Schubert, N. M. Kronenberg, K. Ostermann, S. Hofling, M. C. Gather, An exciton-polariton laser based on biologically produced fluorescent protein. Sci. Adv. 2, e1600666 (2016). 27551686 10.1126/sciadv.1600666 PMC4991930

[39] Y. Wang, P. Shen, J. Liu, Y. Xue, Y. Wang, M. Yao, L. Shen, Recent advances of organic solar cells with optical microcavities. Solar RRL 3, 1900181 (2019).

[40] B. Kippelen, J.-L. Brédas, Organic photovoltaics. Energ. Environ. Sci. 2, 251–261 (2009).

[41] K. A. Mazzio, C. K. Luscombe, The future of organic photovoltaics. Chem. Soc. Rev. 44, 78–90 (2015). 25198769 10.1039/c4cs00227j

[42] G. J. Hedley, A. Ruseckas, I. D. W. Samuel, Light harvesting for organic photovoltaics. Chem. Rev. 117, 796–837 (2017). 27951633 10.1021/acs.chemrev.6b00215 PMC5269644

[43] P. Cheng, G. Li, X. Zhan, Y. Yang, Next-generation organic photovoltaics based on non-fullerene acceptors. Nat. Photonics 12, 131–142 (2018).

[44] E. Hecht, *Optics* (Pearson Education Incorporated, 2017).

[45] D. Wang, H. Kelkar, D. Martin-Cano, D. Rattenbacher, A. Shkarin, T. Utikal, S. Götzinger, V. Sandoghdar, Turning a molecule into a coherent two-level quantum system. Nat. Phys. 15, 483–489 (2019).

[46] C. Gardiner, *Stochastic Methods: A Handbook for the Natural and Social Sciences* (ed. 4, 2009).

[47] J. del Pino, J. Feist, F. J. Garcia-Vidal, Quantum theory of collective strong coupling of molecular vibrations with a microcavity mode. New J. Phys. 17, 053040 (2015).

[48] J. V. Wall, C. R. Jenkins, *Practical Statistics for Astronomers* (Cambridge Univ. Press, 2003).

[49] L. V. Wang, H.-i. Wu, *Biomedical Optics: Principles and Imaging* (John Wiley & Sons, 2012).

[50] K. Yamashita, U. Huynh, J. Richter, L. Eyre, F. Deschler, A. Rao, K. Goto, T. Nishimura, T. Yamao, S. Hotta, H. Yanagi, M. Nakayama, R. H. Friend, Ultrafast dynamics of polariton cooling and renormalization in an organic single-crystal microcavity under nonresonant pumping. ACS Photonics 5, 2182–2188 (2018).

