# Supplementary material for: Superabsorption in an organic microcavity: Toward a quantum battery
Source: Sci Adv. 2022 Jan 14;8(2):eabk3160. doi: 10.1126/sciadv.abk3160 (PMC8759743; doi:10.1126/sciadv.abk3160)
Supplement: Supplementary file 1 — Sections S1 to S4 Tables S1 to S3 Figs. S1 to S17 Legend for movie S1 References [file sciadv.abk3160_sm.pdf]

Science Advances
AAAS

Supplementary Materials for

Superabsorption in an organic microcavity: Toward a quantum battery

James Q. Quach*, Kirsty E. McGhee, Lucia Ganzer, Dominic M. Rouse, Brendon W. Lovett, Erik M. Gauger, Jonathan Keeling, Giulio Cerullo, David G. Lidzey, Tersilla Virgili*

*Corresponding author. Email: quach.james@gmail.com (J.Q.Q.); tersilla.virgili@polimi.it (T.V.)

Published 14 January 2022, *Sci. Adv.* **8**, eabk3160 (2022)
DOI: 10.1126/sciadv.abk3160

The PDF file includes:

Sections S1 to S4
Tables S1 to S3
Figs. S1 to S17
Legend for movie S1
References

Other Supplementary Material for this manuscript includes the following:

Movie S1

S1. CHARACTERISATION OF SAMPLES AND CALIBRATION MEASUREMENTS

This section presents further details of the properties of the fabricated microcavities, and measurements used to calibrate the results in the main text.

a. Quenching at large concentrations Figure S1 shows the photoluminescence quantum yield (PLQY) as a function of dye concentration. At large concentration the yield drops to zero; this provides an upper limit on the concentration that can be studied in experiment. The photoluminescence measurements were taken using a Coherent Mira 900 laser operating at 400 nm with a repetition rate of 80 MHz. The laser beam was focused onto the surface of a sample placed at the centre of an integrating sphere. The laser light and the emission from the sample were scattered by the diffuse interior of the sphere and collected by an optic fibre, which was coupled to an Andor Shamrock SR-303i-A CCD spectrometer. Spectra were taken for different concentration LFO films, as well as a blank glass substrate in order to calculate the proportion of laser light absorbed by the LFO samples.

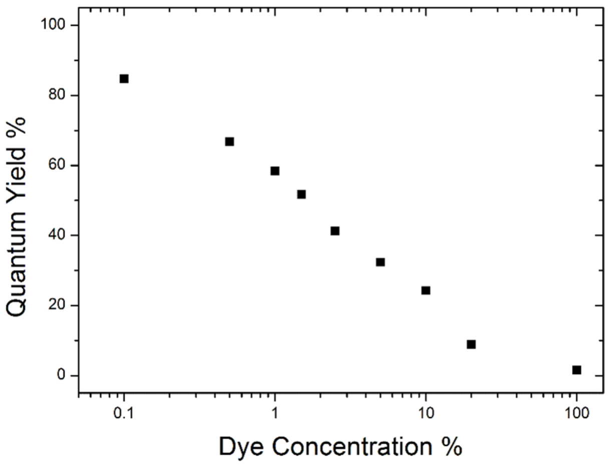

FIG. S1. Photoluminescence quantum yield as a function of LFO concentration.

b. Film and microcavity spectra Figure S2 shows the molecular absorption and emission spectra, and examples of the microcavity reflectivity spectra. Panels (a,b) show the properties of the bare molecular film. These show the small Stokes shift between absorption and photoluminescence, and also show how high film concentrations modify the photoluminescence spectrum, consistent with the reduced PLQY shown above. The microcavity reflectivity spectra (c,d) show the crossover from weak- to strong-coupling as the concentration is increased.

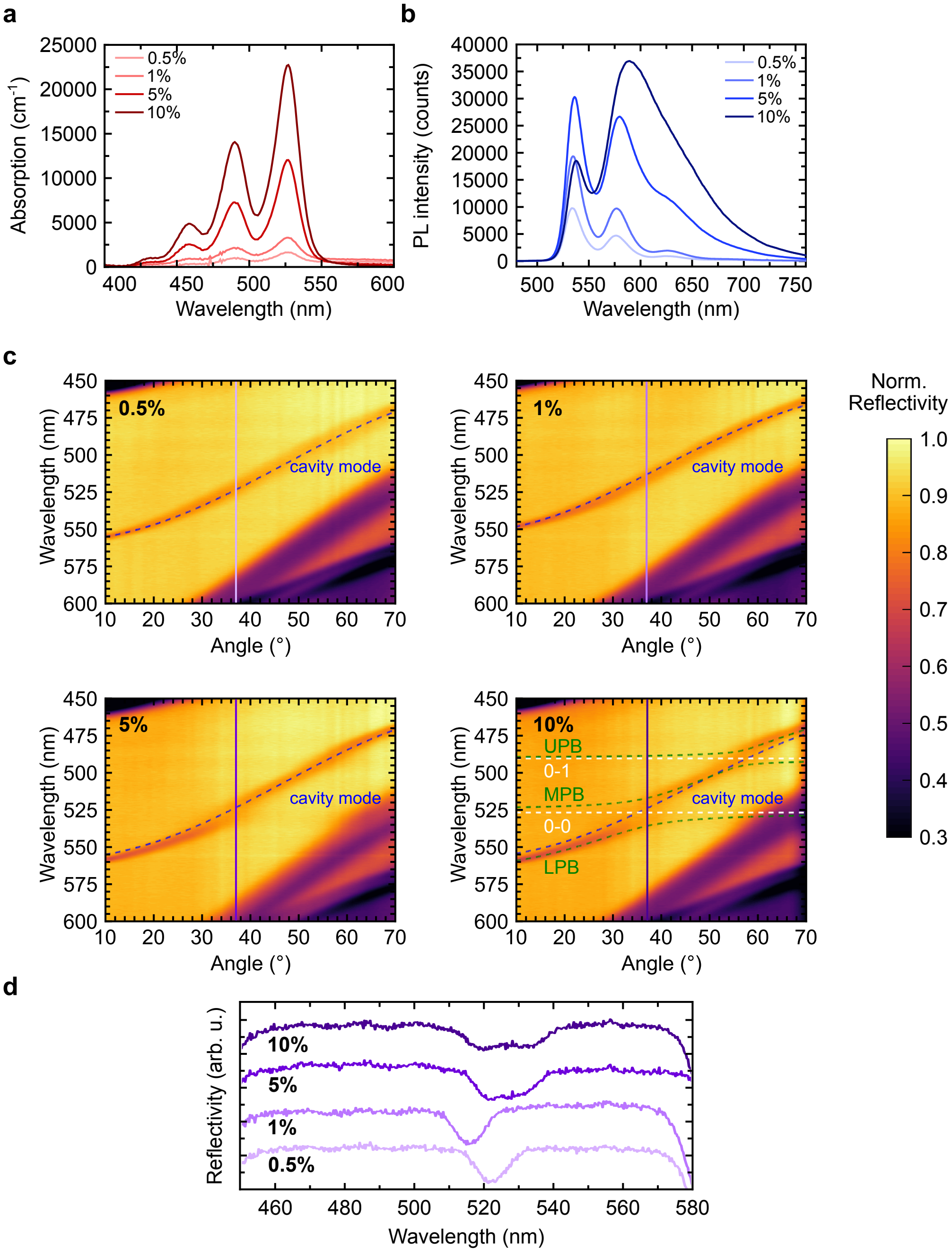

FIG. S2. **Absorption, photoluminescence of the LFO films and reflectivity spectra of the microcavities.** (a) Absorption and (b) photoluminescence spectra for the 0.5%, 1%, 5%, and 10% LFO-concentration films. (c) Reflectivity spectra for 0.5%, 1%, 5%, and 10% LFO-concentration microcavities. UPB, MPB, and LPB label the upper, middle, and lower polariton branches, respectively. Also indicated are the 0-0 and 0-1 transition wavelengths. (d) is a slice of the reflectivity spectra at 37°. The single dip in the 0.5% and 1% concentration spectra indicate the weak-coupling regime. The double dip seen in the 10% concentration spectra, represent the polaritonic states, indicating the strong-coupling regime. The 5% concentration spectrum represents a situation intermediate between a single and double dip, indicating an intermediate-coupling regime.

c. Transfer matrix calculations To provide bounds on the cavity lifetime, separate from the measured cavity linewidth—which contains effects of inhomogeneous broadening—we make use of transfer matrix calculations of the cavity structure. These calculations, shown in Fig. S3, also enable one to visualise the electric field intensity in the microcavity structure. These calculations give a designed cavity lifetime of 306fs, which serves as an upper bound of the actual cavity lifetime.

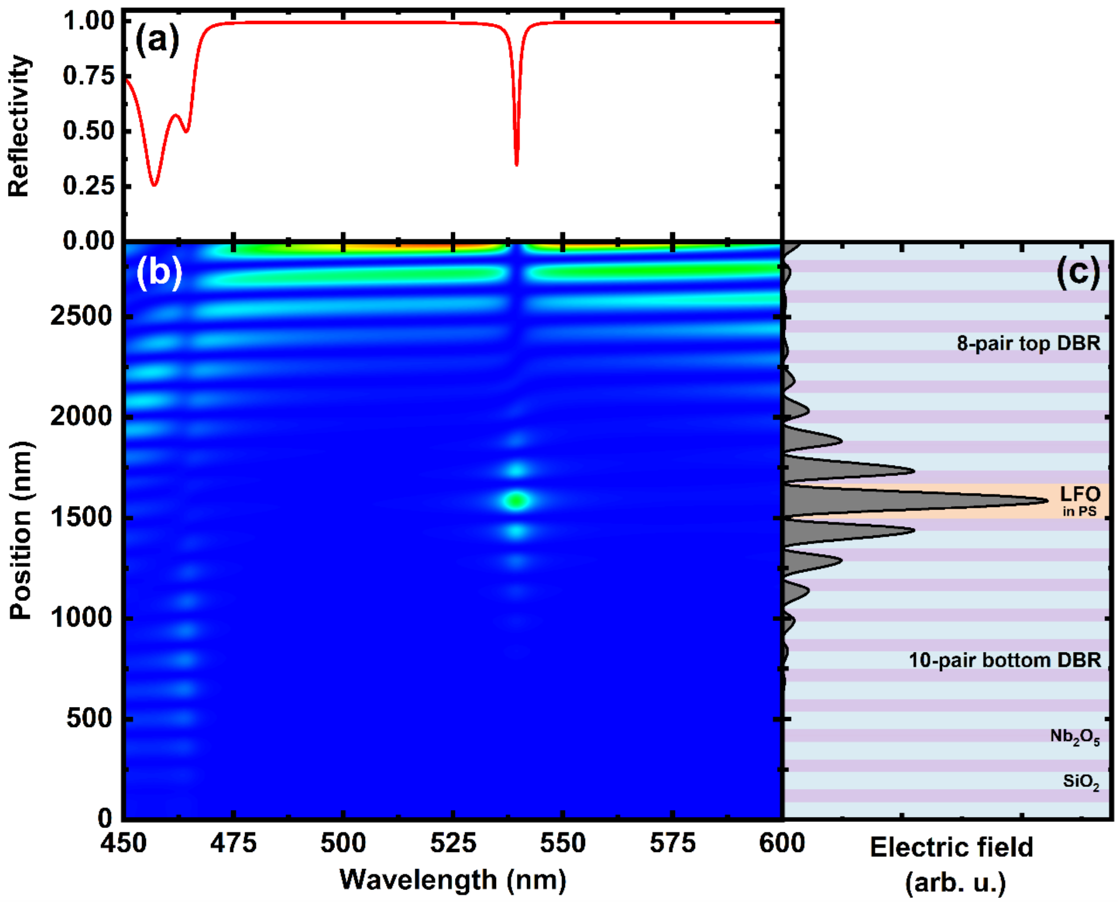

FIG. S3. **Transfer matrix simulation of the electric field distribution for the 1% cavity.** (a) shows the cavity reflectivity, (b) the spectrally-resolved electric field amplitude, and (c) the electric field amplitude at the cavity mode wavelength. The shaded sections of (c) indicate the different materials which make up the cavity, with Nb2O5 in purple (refractive index, n = 2.25), SiO2 in blue (n = 1.52), and LFO in PS in orange (n = 1.60). All simulations were made using transfer matrix modelling at an angle of 20° to the cavity normal to maintain consistency with the transient reflectivity measurements.

d. Pump-probe dynamics of bare films For comparison to the pump-probe dynamics of the cavity shown in the main text, Figs. S4,S5 show the transient transmission spectra of the bare films. (Note that, as discussed in the main text, a transmission geometry is required for transient spectroscopy of the bare films).

Figure S4 compares the dynamics at 525 nm (ground state bleaching) and 571 nm (stimulated emission region) for the 1% concentration film. Unfortunately a coherent artefact, due to the degenerate pump-probe configuration, masks the time dynamics in the first 100 fs. However, the same rise and decay times are seen at both probe wavelengths, indicating that we are probing the same exciton population.

We also explored pump-fluence dependence of the bare films. No dependence on the excitation fluence was detected in any control film. This indicates the absence of bimolecular effects or multi-photon excitation. Figure S5 shows such data for all concentrations, at two different pump fluences. As in Fig. S4, a coherent artefact is present at zero delay due to the degenerate pump and probe spectra.

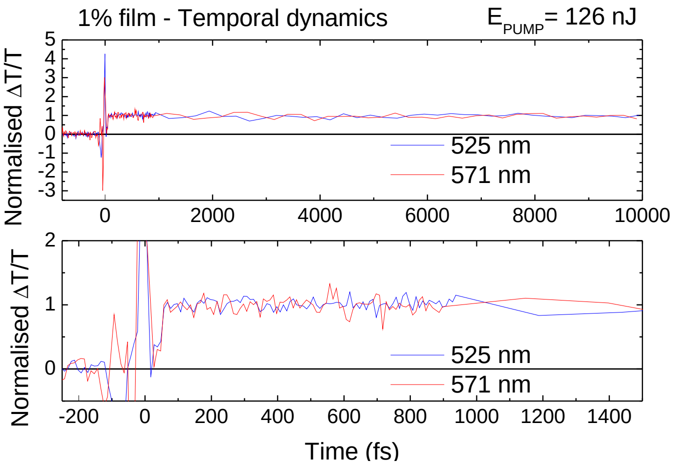

FIG. S4. **Dynamics of the control 1% control film at different wavelengths.** The two panels show two different time windows (top panel until 10 ps, bottom panel until 1.4 ps. The same behavior is observed for other film concentrations.

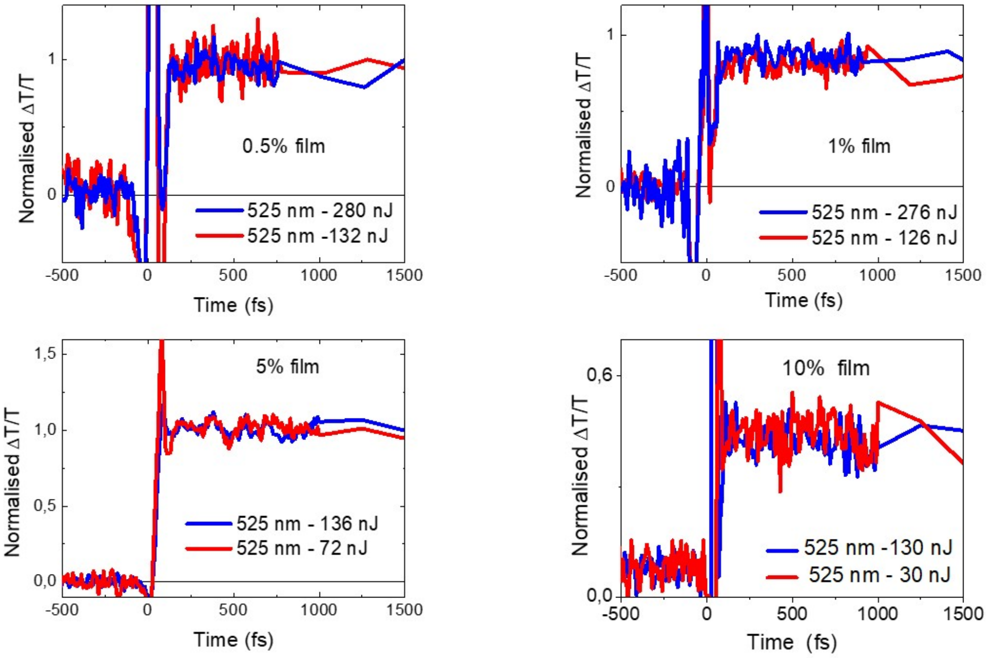

FIG. S5. **Dynamics of the control films, at different concentrations.** Each panel shows the dynamics for a different concentration, as indicated.

e. Estimating number of molecules in film. To determine the number of LFO molecules in the microcavities we study, we first determined the absorption cross-section of a single LFO molecule, σ_{LFO}. The transmission spectrum of a 0.1% solution of LFO in 25 mg/mL PS/dichloromethane in a 1 mm thick cuvette was measured using a Horiba Fluoromax 4 fluorometer with a xenon lamp. The absorption coefficient ($\alpha = n\sigma_{LFO}$) of the 0–0 transition was then calculated using the relation $T/T_0 = e^{-\alpha d}$, where T/T_0 is the fractional transmission of the xenon lamp at the 0–0 transition, d is the cuvette thickness, n is the number density of absorbing molecules in solution per unit volume, and σ_{LFO} is the absorption cross-section of a single LFO molecule [49]. Using the known value of n for this solution, σ_{LFO} was calculated as 3.3×10^{-16} cm^2. The transmission of the 10% LFO concentration in film was then measured to obtain α and hence n (number density of molecules in the cavity active layer), using the measured value of σ_{LFO}, with d (film thickness) measured using a Bruker DektakXT profilometer. This value was then multiplied by the area of the laser beam and d to obtain N. Here we assume a uniform distribution in the active layer. N for other concentrations were scaled accordingly.

f. Estimating number of photons in cavity. To estimate the number of photons entering the cavity in each different cavity, we consider the overlap between the pump spectrum and the cavity transmission. The number of photons in the cavity is given by multiplying the number of pump photons by $1 - R$, where R is the reflectivity of the cavity: $n = N_\gamma(1 - R)$. An example of this is shown in Fig. S6, for the 1% cavity. Starting from the pump spectrum (yellow curve), by considering the reflectivity spectrum of the cavity (black line), we calculate the fraction of photons entering into the cavity (purple line). Table S1 shows the resulting estimates of photon numbers for each experiment.

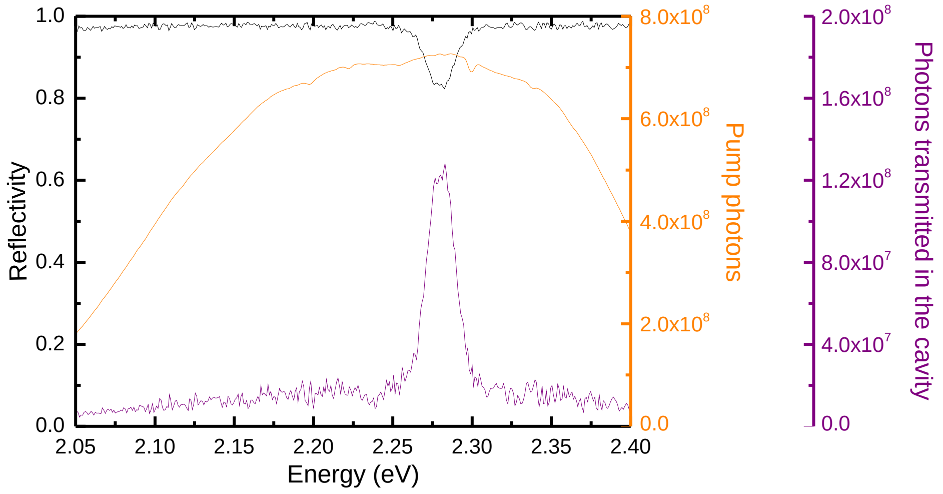

FIG. S6. **Calculating photon number**. Yellow curve (right axis): pump spectrum. Black curve (left axis) reflectivity spectrum of 1% cavity. Purple curve (right axis): photons transmitted into the cavity.

Experiment	$N_{\text{dye}}(\times 10^{10})$	$N_{\text{photon}}(\times 10^{10})$
**A1**	16.20	1.90
**A2**	8.08	0.98
**A3**	1.62	0.26
**B1**	1.62	4.53
**B2**	0.81	0.16

TABLE S1. Estimated photon number and molecule number for each experiment.

g. Derivative features in the differential transmitivity As seen in Fig. 2 of the main text, the transient signal shows both positive and negative features in the differential transmitivity. This can be understood from the existence of a derivative feature in the spectrum. Such a feature occurs if the pump causes an absorption

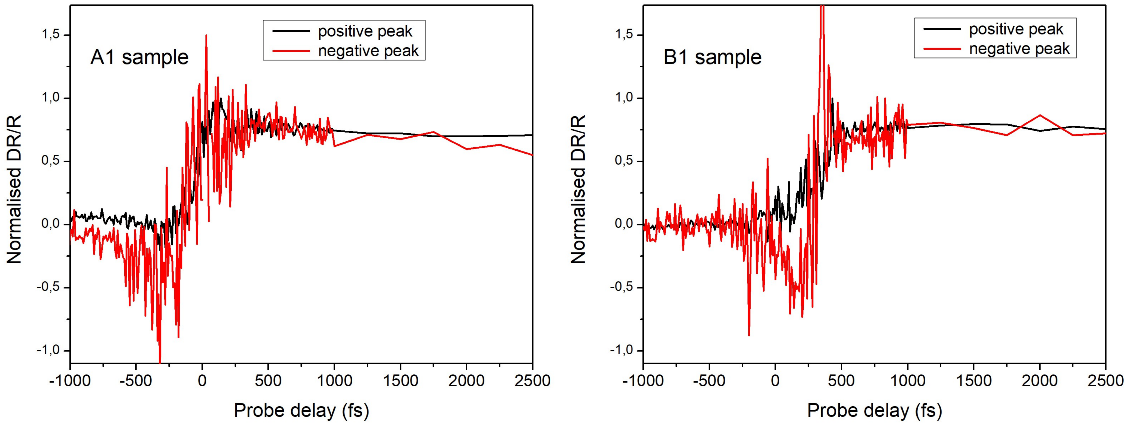

FIG. S7. Time evolution of positive and negative features in the differential transmitivity.

peak to move in energy. In that case the change of absorption will see a decrease in absorption where the feature used to be, and an increase where the feature now is [33,50]. This leads to a feature that takes the form of the derivative of the original absorption peak with respect to energy, and which thus contains both negative and positive contributions. This structure is exactly what we observe, indicating that the feature corresponds to a resonance which moves with excited state population. This feature though coexists with other positive features in the transient reflectivity, arising from mechanisms such as ground state bleaching or stimulated emission. Whether a negative feature is seen depends on how these contributions compete and whether the feature is above the detection threshold.

The presence of this derivative feature does not influence our determination of the energy stored in the molecules and the charging time. As shown in the Fig. S7, the time dependence of the negative and positive peaks is the same. (The negative feature does show more noise, as this signal is weaker compared to background noise.) Since both features show the same time evolution, they would both recover the same theoretical fits.

S2. THEORETICAL MODELLING

In this section we describe our approach to modelling the system. We first provide a more detailed discussion of the model we use, and the physical origin of the terms involved. We then derive the equations of motion for the expectation values using a second-order cumulant approach.

A. Details of theoretical model

As discussed in the main text, we model the experiments through a Dicke model—which describes N two-level systems coupled to a photon mode. Describing organic molecules as two-level systems is an approximation, as it neglects both the role of rotational and vibrational molecular modes, as well as the existence of higher excited states of the molecules. It is known that this approximation can become valid in some limiting situations, such as at low temperatures [45]. While our experiments are performed at room temperature, we nonetheless expect the model to provide a reasonable approximation. This is because, as noted in the main text, the molecules we consider show a small Stokes shift, indicating vibrational dressing of optical transitions is weak. Moreover, as seen in the main text and discussed further below, our model matches the experimental results well without such additional features.

The Hamiltonian describing our system, including the external pump laser, takes the form (setting $\hbar = 1$):

$$H = \Delta_c a^\dagger a + \sum_{j=1}^{N} \left[\frac{\Delta_a}{2} \sigma_j^z + g(a^\dagger \sigma_j^- + a\sigma_j^+) \right] + i\eta(t)(a^\dagger - a)\,, \tag{1}$$

where a ($a^\dagger$) is the photon annihilation (creation) operator, σ_i^α for $\alpha = x, y, z$ are the Pauli matrices. We write the Hamiltonian in the rotating frame of the pump laser, so Δ_a (Δ_c) is the energy detuning of the laser from the cavity (molecules), g is the coupling strength of each molecule to the photon mode, $\eta(t)$ is the Gaussian profile of the pump laser. In the following we assume that the molecules and cavity are resonant ($\Delta_a = \Delta_c \equiv \Delta$) and that $\Delta = 0$ unless specifically noted.

To account for dissipative processes, we consider the time evolution of the density matrix, including Lindblad terms for various dissipative processes:

$$\dot{\rho} = -i[H, \rho] + \kappa \mathcal{L}[a] + \sum_{j=1}^{N} \left(\gamma^z \mathcal{L}[\sigma_j^z] + \gamma^- \mathcal{L}[\sigma_j^-] \right)\,, \tag{2}$$

where $\mathcal{L}[X] = X\rho X^\dagger - (1/2)\{X^\dagger X, \rho\}$. The first term, with rate κ, describes loss of photons, due to the finite reflectivity of the cavity mirrors. The second term, with rate γ^z, describes dephasing of the molecular excitations. This term describes the effect of coupling between the molecular electronic state and vibrational degrees of freedom—vibrations both of the molecule and of the polystyrene matrix in which it is contained. The final term, γ^-, describes the decay of electronic excitation, due to emission into non-cavity modes, such that $1/\gamma^-$ would be the excited state lifetime in the absence of the cavity.

As noted in the main text, dephasing γ^z plays a crucial role in the dynamics, with quite distinct behaviour occurring with and without dephasing. In particular, dephasing introduces transitions between the "bright" and "dark" molecular excited states. To understand these states, let us first consider states with a single excitation. The form of Eq. (1) shows that the cavity photon couples only to the totally symmetric molecular excited state, i.e. a state with equal weight and phase of excitation on all molecules. However, for N molecules, N excited states exist. The remaining $N - 1$ states are orthogonal to the coupling to light, and are known as "dark states". While we have introduced this for the space with a single excitation, a generalization to higher excited states exists. In this case, the language of superradiant and subradiant states is often used [1], with superradiant states referring to those that can be reached using the collective raising and lowering operators, $\sum_j \sigma_j^\pm$. Because the dephasing term acts on individual molecules, it describes a process that allows loss of phase coherence between different molecules. As such, this causes a transition from the optically bright state created by the laser, to an incoherent mixture of bright and dark states. Since the dark states do not couple to the cavity, this process is responsible for the asymmetry between collectively enhanced absorption, and the lack of collective enhancement of emission.

For N identical molecules of energy ω_a (in the lab frame), the energy density stored on the molecules is given by

$$E(t) = \frac{\omega_a}{2}\left(\langle\sigma^z(t)\rangle + 1\right) . \tag{3}$$

As such, in the following, our aim is to predict the time evolution of this quantity.

B. Cumulant equations

To determine the time evolution of $\langle\sigma^z(t)\rangle = \mathrm{Tr}\left[\sigma^z\rho(t)\right]$ we begin by writing down the first order expectation values of the system. We adopt the notation $C_a(t) \equiv \langle a(t)\rangle$ for photon operators, and $C_{\alpha=x,y,z}(t) \equiv \langle\sigma^\alpha(t)\rangle$ for spin operators, along with a similar notation for higher order expectations, e.g., $C_{ax}(t) \equiv \langle a\sigma^x(t)\rangle$. The equations of motion for the first order expectation values are

$$\partial_t C_a = -\left(i\Delta_c + \tfrac{1}{2}\kappa\right) C_a - \tfrac{1}{2} g N \left(iC_x + C_y\right) + \eta(t) , \tag{4}$$

$$\partial_t C_x = -\Delta_a C_y - 2g\mathrm{Im}\left[C_{az}\right] - \gamma^{\mathrm{tot}} C_x , \tag{5}$$

$$\partial_t C_y = \Delta_a C_x - 2g\mathrm{Re}\left[C_{az}\right] - \gamma^{\mathrm{tot}} C_y , \tag{6}$$

$$\partial_t C_z = 2g\left(\mathrm{Re}\left[C_{ay}\right] + \mathrm{Im}\left[C_{ax}\right]\right) - \gamma^-\left(C_z + 1\right) , \tag{7}$$

where ∂_t is short for $\frac{\partial}{\partial t}$, $\gamma^{\mathrm{tot}} = 2\gamma^z + \frac{1}{2}\gamma^-$, and for notational ease we have dropped the explicit time dependence of observables. As described in the main text, in mean field theory we would now set the second order cumulants to zero. These are defined as

$$\langle\langle AB\rangle\rangle = \langle AB\rangle - \langle A\rangle\langle B\rangle . \tag{8}$$

This would result in the usual decomposition of second order expectation values into products of first order ones, $C_{AB} = C_A C_B$, which is the assumption that molecule-molecule, molecule-photon, photon-photon and all higher order correlations are negligible. However, we instead derive equations of motion for the second order expectation values, capturing the leading order $1/N$ corrections to mean field theory. The second order photon correlations obey:

$$\partial_t C_{a^\dagger a} = -\kappa C_{a^\dagger a} - gN\left(i\mathrm{Im}\left[C_{ax}\right] + \mathrm{Re}\left[C_{ay}\right]\right) + 2\eta(t)\mathrm{Re}\left[C_a\right] , \tag{9}$$

$$\partial_t C_{aa} = -\left(2i\Delta_c + \kappa\right) C_{aa} - gN\left(iC_{ax} + C_{ay}\right) + 2\eta(t) C_a , \tag{10}$$

while molecule-photon correlations follow:

$$\begin{aligned}\partial_t C_{ax} = &-\left(i\Delta_c + \tfrac{1}{2}\kappa + \gamma^{\mathrm{tot}}\right) C_{ax} - \Delta_a C_{ay} - i\frac{g}{2}\left[1 + (N-1)\right] C_{xx} \\ &- \frac{g}{2}\left[iC_z + (N-1) C_{xy}\right] + ig\left(C_{aaz} - C_{a^\dagger az}\right) + \eta(t) C_x ,\end{aligned} \tag{11}$$

$$\begin{aligned}\partial_t C_{ay} = &-\left(i\Delta_c + \tfrac{1}{2}\kappa + \gamma^{\mathrm{tot}}\right) C_{ay} + \Delta_a C_{ax} - i\frac{g}{2}\left[-iC_z + (N-1) C_{xy}\right] \\ &- \frac{g}{2}\left[1 + (N-1) C_{yy}\right] - g\left(C_{aaz} + C_{a^\dagger az}\right) + \eta(t) C_y ,\end{aligned} \tag{12}$$

$$\begin{aligned}\partial_t C_{az} = &-\left(i\Delta_c + \tfrac{1}{2}\kappa\right) C_{az} - \gamma^-\left(C_{az} + C_a\right) - \frac{g}{2}\left[-iC_x + (N-1) C_{yz}\right] \\ &- i\frac{g}{2}\left[iC_y + (N-1) C_{xz}\right] + g\left(C_{aay} + C_{a^\dagger ay}\right) - ig\left(C_{aax} - C_{a^\dagger ax}\right) + \eta(t) C_z .\end{aligned} \tag{13}$$

These now depend on third order expectation values, some of which contain multiple Pauli operators. We must note that these terms indicate Pauli operators representing different molecules and so commute — we have already taken into account the cases where the Pauli operators correspond to the same molecule by using the Pauli algebra $\sigma^\alpha\sigma^\beta = \mathbb{1}\delta^{\alpha\beta} + i\sigma^\gamma\epsilon^{\alpha\beta\gamma}$. The molecule-molecule expectation values for the same Pauli operator acting on different molecules are

$$\partial_t C_{xx} = -2\Delta_a C_{xy} - 4g\mathrm{Im}\left[C_{axz}\right] - 2\gamma^{\mathrm{tot}} C_{xx} , \tag{14}$$

$$\partial_t C_{yy} = 2\Delta_a C_{xy} - 4g\mathrm{Re}\left[C_{ayz}\right] - 2\gamma^{\mathrm{tot}} C_{yy} , \tag{15}$$

$$\partial_t C_{zz} = 4g\left(\mathrm{Im}\left[C_{axz}\right] + \mathrm{Re}\left[C_{ayz}\right]\right) - 2\gamma^-\left(C_{zz} + C_z\right) . \tag{16}$$

Finally, the molecule-molecule expectation values for different Pauli operators acting on different molecules are

$$\partial_t C_{xy} = \Delta_a (C_{xx} - C_{yy}) - 2g (\mathrm{Re}[C_{axz}] + \mathrm{Im}[C_{ayz}]) - 2\gamma^{\mathrm{tot}} C_{xy} , \tag{17}$$

$$\partial_t C_{xz} = -\Delta_a C_{yz} + 2g (\mathrm{Re}[C_{axy}] + \mathrm{Im}[C_{axx}] - \mathrm{Im}[C_{azz}]) - \gamma^{\mathrm{tot}} C_{xz} - \gamma^- (C_{xz} + C_x) , \tag{18}$$

$$\partial_t C_{yz} = \Delta_a C_{xz} + 2g (\mathrm{Re}[C_{ayy}] - \mathrm{Re}[C_{azz}] + \mathrm{Im}[C_{axy}]) - \gamma^{\mathrm{tot}} C_{yz} - \gamma^- (C_{yz} + C_y) . \tag{19}$$

In principle one can continue to write equations of motion for increasingly higher orders of expectation values, however, at large N, most essential physics is obtained at second order. We therefore truncate the cumulant expansion by setting third order cumulants to zero. These are defined as

$$\langle\langle ABC \rangle\rangle = \langle ABC \rangle - \langle AB \rangle\langle C \rangle - \langle A \rangle\langle BC \rangle - \langle AC \rangle\langle B \rangle + 2\langle A \rangle\langle B \rangle\langle C \rangle , \tag{20}$$

and so setting $\langle\langle ABC \rangle\rangle = 0$ closes the system of differential equations, allowing us to write $\langle ABC \rangle$ in terms of first and second order correlations.

C. Behaviour in the thermodynamic limit

In Fig. S8 we present the theoretical N-dependence of the charging time τ, maximum energy density E_{max}, and maximum power density P_{max} over a wider range of N than shown in Fig. 3 of the main text. This shows that in addition to the decay-dominated (purple) and coupling-dominated (green) behavior described in the main text, a third region occurs at even larger N, which we discuss below.

In the main text we discussed the energetic dynamics around the decay-to-coupling dominated crossover regime, as this was the experimental operating region. Moving deeper into coupling-dominated regime does not necessarily improve the energy storage properties. This is illustrated in Fig. S8(b) which shows the simulated four points, corresponding to the circles in Fig. S8(a). Within the coupling-dominated regime, energy stored within the microcavity rapidly oscillates which is not a desirable feature. This occurs because the light and matter degrees of freedom hybridise to form polaritons with upper and lower branches split by Rabi frequency $\pm g\sqrt{N}$, leading to beating between these modes. These oscillations are not present in the experimentally studied crossover region. In this region, dephasing is strong enough to prevent oscillation in energy, yet weak enough to warrant superextensive charging. Therefore, this is the optimal region to produce a QB. Going deeper into the coupling-dominated regime would only be advantageous if energy was extracted on a shorter timescale than the period of oscillations, or additional mechanisms were in place to stabilise the oscillations.

At even larger N (red region) the stored energy falls with increasing N. This can be understood as arising from a condition where the polariton energy splitting exceeds the bandwidth of the pump (set by its finite pulse duration), suppressing energy absorption. Numerically, we find this occurs when $N > N_\sigma$ where $g\sqrt{N_\sigma} = (2/5)^{\frac{1}{4}} (1/\sigma)$, which signifies the onset of this non-resonant regime. The prefactor $(2/5)^{\frac{1}{4}}$ will be explained in Section S2 D. To build an efficient QB in this regime, one should tune the frequency of the laser to match the polariton energies. Additionally, the time dynamics of energy absorption here change significantly, with the second half of the laser pulse causing stimulated emission, reducing the stored energy — such dynamics arises naturally from a toy model of strongly coupled modes with a splitting larger than the pulse bandwidth, and can be seen in the form of the red line in Fig. S8(b).

In Figure S9, we show the theoretical N-dependence of τ, E_{max} and P_{max} when the frequency of the driving laser is tuned resonant to the lower polariton, i.e. $\Delta_a = \Delta_c = g\sqrt{N}$ in the cumulant equations given in Section S2 B. We emphasise that this is not the condition under which the experiments were performed, but of theoretical interest. By comparison of Figures S9 and S8 one can see that the behaviour of the measures with the different driving frequencies are the same until $N > N_\sigma$. In Figure S9 when $N > N_\sigma$, the laser frequency continues to drive at the frequency of the lower polariton, instead of at the molecular energy as in Figure S8. In this case, the total energy and power in the cavity continue to grow linearly with N, and so the energy and power densities are constant.

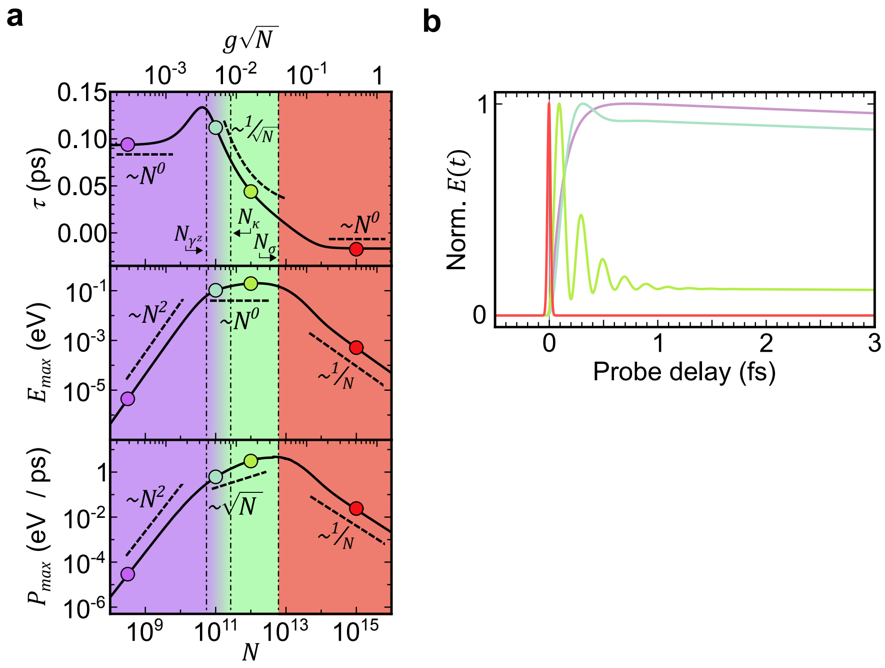

FIG. S8. **Charging dynamics vs number of molecules N.** (a) Charging time, peak stored energy, and maximum power as a function of N. This figure is identical to Fig. 3(a) in the main text, but extended to larger range of N. At large N the stored energy can reach half maximum before the laser pulse finishes, in which case the charging time τ becomes negative. (b) Examples of dynamics in each regime. The values of N used in these dynamics corresponds to the circles of the same colour in (a).

D. The boundary between decay and coupling dominant regimes

There are two timescales in this system: the vacuum Rabi splitting (i.e. polariton detuning) $g\sqrt{N}$ and the Rabi splitting $g\sqrt{rN}$ where rN is the number of photons in the cavity. In Fig. 3(a), we show that the microcavity charges super-extensively once $g\sqrt{N}$ is greater than all decay channels. However, this is only true if $r \leq 1$, as is true in experiments A1, A2 and A3. In Fig. 3(b), the boundaries N_κ and N_{γ^z} are instead determined by $g\sqrt{rN}$ being equal to the decay rates. This is because $r \geq 1$ in experiments B1 and B2. More generally, the important timescale is the larger of the polariton detuning and the Rabi splitting, and so the coupling dominant regime occurs when $g\sqrt{\mathrm{Max}(1,r)N}$ is larger than all decay channels.

In Figure S10 we plot the charging time τ as a function of N and r. Here, we set $\kappa = \gamma^- = \gamma^z \equiv \Gamma = 2$ meV (note that γ^z is independent of N) so that there is only one boundary between the decay dominant and coupling dominant regimes. The green, red and dashed-black lines show the boundaries between the decay dominant and coupling dominant regimes ($N = N_\Gamma$) if $g\sqrt{N}$, $g\sqrt{rN}$ or $g\sqrt{\mathrm{Max}(1,r)N}$ are used as the relevant coupling scale respectively. Clearly, the boundary is determined by $g\sqrt{\mathrm{Max}(1,r)N}$ for all values of r. We also show the boundary between the coupling dominant and non-resonant regimes ($N = N_\sigma$) as the cyan line. When $r > 1$, we find that N_σ becomes linearly dependent on r. The prefactor $(2/5)^{\frac{1}{4}}$ is necessary for N_σ to align with the contours of increased charging time for $r > 1$.

E. Dependence on laser intensity

Figure S11 shows how capacity, charging time and power vary as a function of laser intensity r at fixed number of molecules N. For small r, we find that the maximum energy and power densities vary linearly with r, while charging time is constant. This simply reflects the total energy in the cavity. The charging time is constant because decay channels still dominate over coherent dynamics. As r is increased beyond $r = 1$, the important timescale $g\sqrt{\max(1,r)N}$ begins to scale with r, and so the boundaries separating

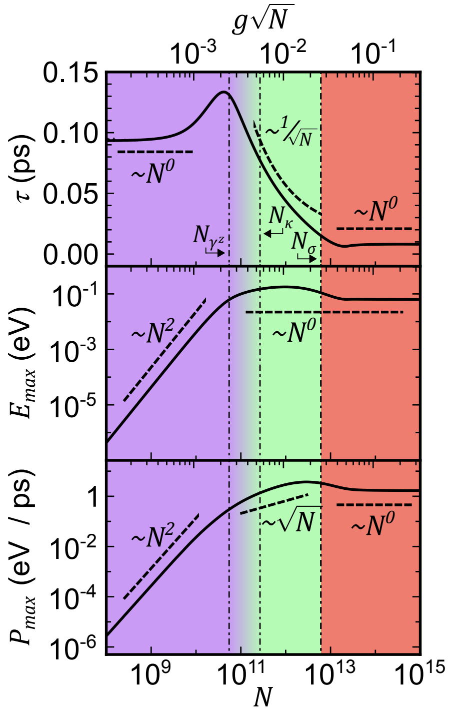

FIG. S9. **Charging dynamics when pumping at lower polariton.** Charging time, peak stored energy, and maximum power as a function of N. This figure is identical to Fig. S8 except that the frequency of the laser is tuned to the lower polariton energy, $\Delta_a = \Delta_c = g\sqrt{N}$, rather than the molecular energy.

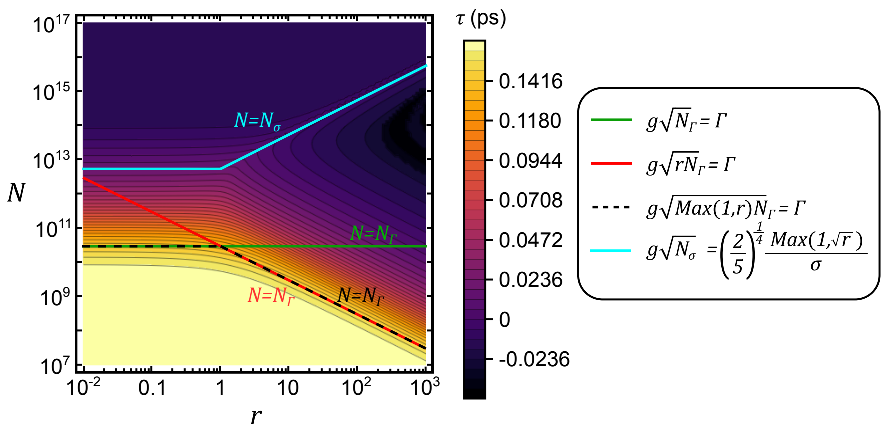

FIG. S10. **Charging time as a function of number of molecules N and laser intensity r.** All parameters are equivalent to the Q1% cavity (see main text) with the exception that the dephasing and non-radiative decay rates are equal to the cavity leakage rate (set to $\gamma^z = \kappa = \Gamma = 2$ meV) and note that the dephasing rate is independent of N.

the coupling dominant and decay dominant regions N_κ and N_{γ^z} are pushed to smaller N. When these boundaries become smaller than the number of molecules in the cavity, the charging time begins to scale as $1/\sqrt{r}$. Additionally, the energy density begins to saturate because there are already many more photons than there are molecules within the cavity. In Figure S11(b) we also plot the experimentally measured energy densities, and we see there is good agreement to the theoretical curve. The coloured points in Figure S11(a) indicate the charging time, maximum capacity and maximum power of the temporal dynamics of the same colour in Figure S11(b).

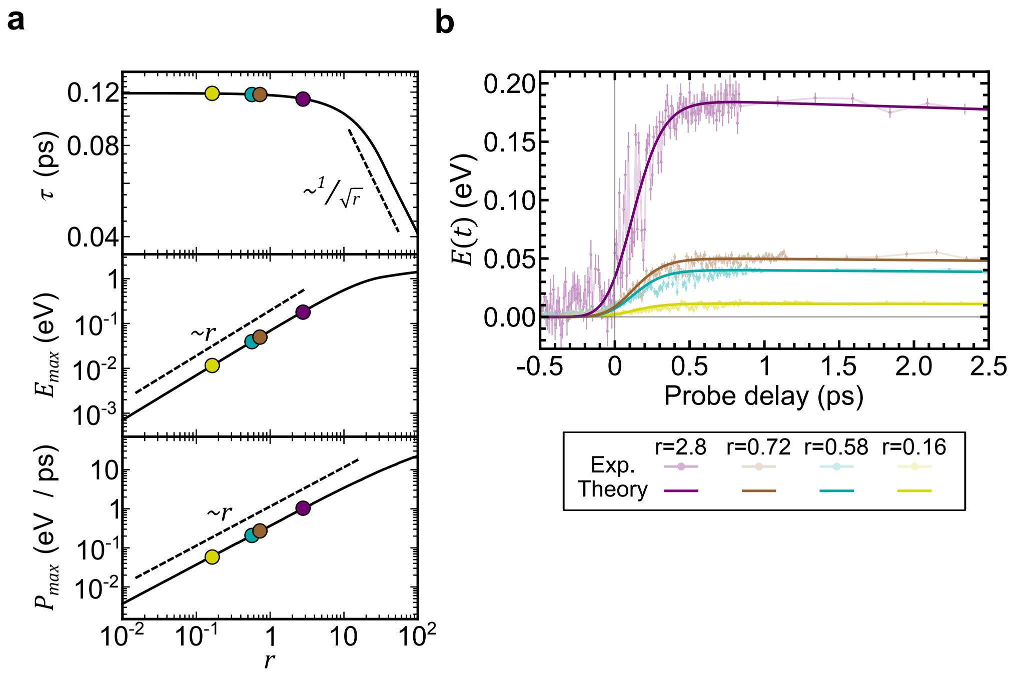

FIG. S11. **Charging dynamics vs pump intensity** r**.** (a) Capacity, charging time and maximum power vs r for a fixed number of molecules $N = 1.62 \times 10^{10}$ (the 1% cavity). All other parameters align with those used to model the 1% cavity. (b) Comparison of theoretical and experimental charging dynamics for four values of r. These are indicated by circles in the charging time panel of (a), showing the experimentally measured values.

S3. FITTING OF MODEL PARAMETERS

As outlined in the main text, we used a reduced chi-squared optimisation procedure to determine the light matter coupling g, dephasing constant γ_0^z and non-radiative decay rate γ^-, as well as to estimate uncertainties on these parameters. As these quantities represent molecular properties, we would expect them to be the same in all the different experiments. For this reason, we performed a global fit rather than performing the procedure individually for each experiment. In this section we give further details on this calculation. As also discussed in the main text, our fitting process is key in estimating the charging time, stored energy and peak charging power. Extracting these quantities requires a smooth curve of charge vs time. Any smoothing process implicitly introduces an effective fitting function (such as e.g. fitting the data to piecewise cubic splines). Since our best guess for such a fitting function is in fact the theoretical model described above, we use this continuous function, fit to the experimental data, to extract charging times, energies, and powers.

In performing our fitting, it is necessary to use the values of the molecule numbers, estimated as discussed above. Because of this, errors in the estimates of N affect the fitting parameters, and thus the resulting charging timescales and powers. These correlated errors make it challenging to estimate errors in the power-law scaling of charging time vs N.

The cavity lifetime (or equivalently the cavity linewidth κ) can also be considered as a fitting parameter, however the value of this parameter is more strongly constrained by other measurements. As noted above, transfer matrix simulations on the designed cavity give an upper bound of 306fs. An alternate estimate is provided by comparing the theoretical and measured reflectivity spectra of the cavities. As discussed below, this implies a cavity lifetime of 120fs. In the following, we first present fitting results for a cavity lifetime of 120fs, and then in Sec. S3 B we discuss how the results change with alternate cavity lifetimes in the range 120fs to 306fs.

A. Fitting procedure

The steps for our fitting procedure are as follows:

1. Calculate the theoretical $E(t)$ curves for a grid of parameter values g, γ^z, γ^-, along with the values of N relevant for all five experiments, A1, A2, A3, B1 and B2. Based on previous observations, we chose the search region of the parameter space as $g \in [0.1, 5000]$ neV; $\gamma_0^z \in [0.1, 5000]$ meV and $\gamma^- = [0.001, 1]$ meV. Subsequent refinements of this search region were made to give higher resolution near the optimal point.

2. We estimate uncertainties, σ_i on each experimental data point (transient reflectivity vs time), by considering the point-to-point variation. Because the uncertainty is higher near $t = 0$, when the pump arrives, we use different error estimates in different time windows. Specifically, we divide experiments A1 and A2 into five windows $t < -300\text{fs}; -300\text{fs} < t < 300\text{fs}; 300\text{fs} < t < 700\text{fs}; 700\text{fs} < t < 1000\text{fs}; t > 1000\text{fs}$. For experiments A3, B1 and B2 we found that four windows $t < -300\text{fs}; -300\text{fs} < t < 300\text{fs}; 300\text{fs} < t < 1000\text{fs}; t > 1000\text{fs}$ was sufficient. In each window, the uncertainty estimate for each experiment is taken from the variance over a narrow range of points (typically 150fs) where there is no strong time dependence.

3. For each set of parameters, we performed an "internal" chi-squared minimisation to find the optimal scaling factor S between the stored energy $E(t)$ and the measured differential reflectivity $\Delta R/R$, and a time shift between the theory and experiment T_0. That is, we minimise

$$\chi^2 = \sum_i \left[\frac{S \times (\Delta R/R)_i - E(t_i + T_0)}{\sigma_i} \right]^2, \tag{21}$$

with respect to S and T_0. We treat the result of this minimisation as the chi-squared value which we use in the following steps to estimate the meaningful parameters g, γ^z, γ^- and their uncertainties.

Estimating the scaling factor S from first principles is difficult because of reflections by the cavity mirror, hence this factor is found by the best fit value. The time shift reflects uncertainty of delays in the optics, so that it is not a-prior clear when the peak of the pump pulse arrives. After this shift, we define $t = 0$ as the moment the pump arrives. This is important when calculating the charging time τ, which we defined as the time from the arrival of the pump until reaching half maximum energy.

4. We then use the chi-squared value described above, and divide by the total number of degrees of freedom $k_{\text{eff}} = k - 3$ (where k is the total number of data points), to arrive at the final reduced chi-squared $\tilde{\chi}^2$ map. A slice of this three dimensional reduced chi-squared map for the 120 fs lifetime is shown in Figure S12 for $\gamma^- = 0.0263$ meV, which is the optimal non-radiative decay rate given in the main text. The optimal parameter set used in the main text that optimises $\tilde{\chi}^2$ is shown as the red point in Figure S12. We find $\tilde{\chi}^2_{min} = 3.048$, suggesting our estimated measurement uncertainties on $\tilde{\chi}$ are reasonable, but likely underestimates.

5. Finally, the 68% confidence interval for each parameter was estimated by considering the contour for which $\tilde{\chi}^2 = \tilde{\chi}^2_{min} + \frac{1}{k_{\text{eff}}}\Delta^*$ where $\Delta^* = 3.51$ is extracted from the reduced chi-squared distribution for 3 parameters and error tolerance (68%), see [48]. In the right panel of Fig. S12 we show the contour as a white line, and the actual parameter values which lie within this 68% contour as black points.

B. Fitting cavity lifetime

Figure S13 shows the optimal reduced chi-squared as a function of cavity lifetime, along with the corresponding best-fit values of the parameters g, γ_0^z and γ^-, following the fitting procedure described above. The minimal reduced chi-squared is 2.803 occurring for a lifetime of 185 fs.

As noted earlier, the cavity lifetime is also constrained by the measured reflectivity spectrum, shown in Figure S2(d). To check this consistency, Fig. S14 shows the calculated absorption spectra for the 0.5%, 1%, 5% and 10% cavities using the optimal parameter sets given by the 185 fs and 120 fs lifetimes. These are

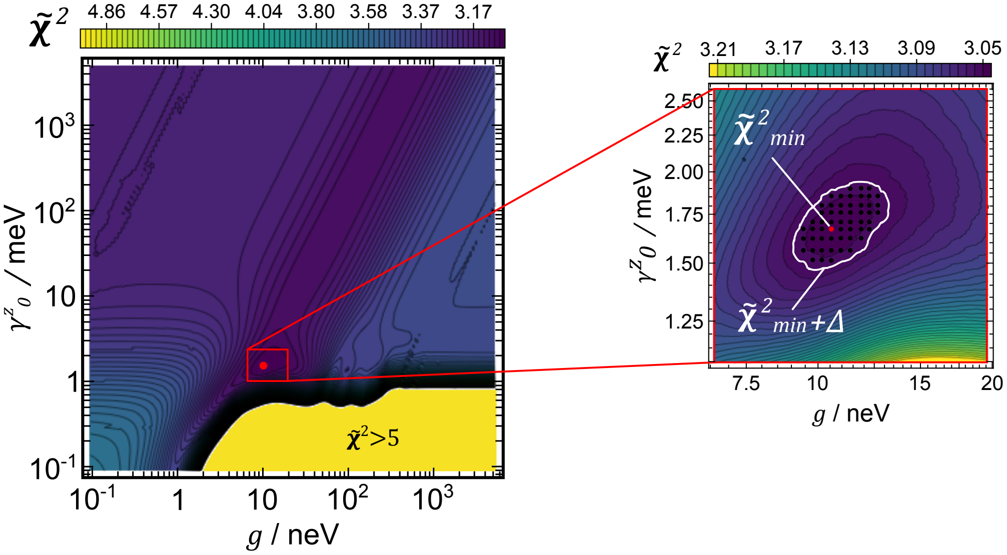

FIG. S12. **Reduced-chi square map to find the optimal parameters for the theoretical model and their 68% confidence intervals.** The chi-squared contour plots shown in this figure are slices of the full three-dimensional map at the optimum non-radiative decay rate $\gamma^- = 0.0141$ meV used in the main text for a cavity lifetime of 120 fs. In the yellow region of the bottom right corner $\tilde{\chi}^2 > 5$, which we do not show to emphasise smaller variations in $\tilde{\chi}^2$. In the right panel, the highlighted contour shows the 68% confidence interval, found using $\Delta = \Delta^*/k_{\text{eff}}$.

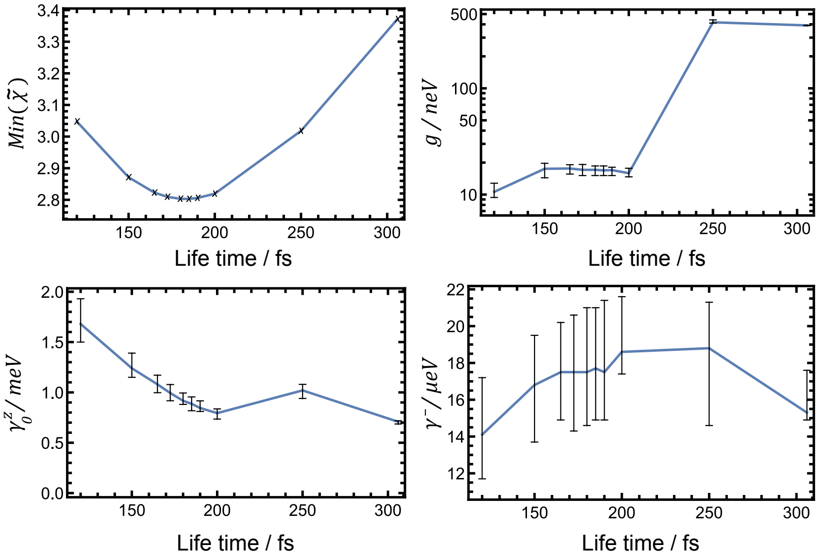

FIG. S13. **Optimal reduced chi-squared, and associated best-fit parameters as a function of cavity lifetime.** Error bars indicate 68% confidence intervals, calculated through the procedure described in Section S3 A.

calculated as $\text{Abs}(\Delta\nu) = \text{Re}\int_0^\infty \exp[i(\Delta\nu+\omega)t]\langle a(t)a^\dagger(0)\rangle$ where $\Delta\nu$ is the energy detuning from the cavity and molecules. When evaluated from our model using the quantum regression theorem, we find

$$\text{Abs}(\Delta\nu) = -\text{Re}\left[\frac{i\Delta\nu - \gamma^{\text{tot}}}{\left(i\left[\Delta\nu + \Omega_{\text{eff}}\right] - \frac{2\gamma^{\text{tot}}+\kappa}{4}\right)\left(i\left[\Delta\nu - \Omega_{\text{eff}}\right] - \frac{2\gamma^{\text{tot}}+\kappa}{4}\right)}\right], \tag{22}$$

where $\Omega_{\text{eff}} = \sqrt{g^2N - (\kappa - 2\gamma^{\text{tot}})^2/4}$ is the effective Rabi splitting. From the measured spectra in Figure S2(d), we expect the 0.5% and 1% cavities to show no polariton splitting, the 5% cavity to have a small

splitting, and the 10% cavity to clearly show strong coupling.

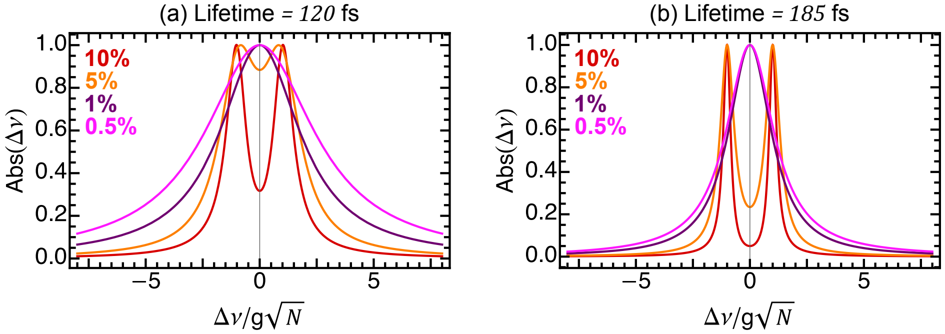

FIG. S14. **Absorption spectra for the** 0.5%**,** 1%**,** 5% **and** 10% **cavities**. The spectrum is calculated using Eq. (22) for the best-fit parameters (see Figure S13) for both 120 fs and 185 fs cavity lifetimes. In the 5% and 10% cavities, there are clear polariton peaks forming at $\Delta\nu = \pm g\sqrt{N}$.

It is clear from Figure S14 that although the 185 fs cavity lifetime gives a smaller reduced chi-squared, these parameters predict significantly stronger coupling than is seen in experimental reflectivity spectra. In contrast, the 120 fs cavity lifetime parameters reproduce the polariton splittings across all cavities more accurately, while showing a reduced chi-squared that is not significantly larger. We therefore conclude that 120 fs cavity lifetime, with g, γ_0^z and γ^- given in the main text provides the best fit to the experimental data. In Table S2, we summarise the scaling factors S and time shifts T_0 that relate the measured differential reflectivity $\Delta R/R$ to the theoretically calculated stored energy $E(t)$, as used to calculate the fits plotted in Figure 2(b) of the main text. For a formal defintion of S, T_0, see Eq. (21).

Experiment	Scaling factor, S	Time shift, T_0/ fs
**A1**	2.32	47.4
**A2**	2.01	-47.4
**A3**	2.93	-140.0
**B1**	3.75	-159.8
**B2**	6.24	-210.5

TABLE S2. **The optimal scaling factors and time shifts used to calculate the theoretical curves in Figure 2(b) in the main text.**

C. Results of fitting procedure

The theoretical time evolution arising from using the above fitting procedure is shown in Figure 3 of the main text. In plotting that figure, the results of the theoretical fit are convolved with an instrument response function, as required to match the experimental data. Figure S15 shows the same data but without convolution by the instrument response, thus providing a more direct picture of the intrinsic dynamics of the system. From the theoretical curves, one can extract the rise time of stored energy τ, the peak stored energy $E_{\max}$, and maximum charging power $P_{\max}$. These values are summarised in Table 1 in the main text.

As well as the scaling to the observables with N shown in Figure 3 in the main text, we could also estimate an effective power-law scaling of the observables $q_i \in \{\tau, E_{\max}, P_{\max}\}$ directly from pairs of experiments i, j by the relation $q_i/q_j = (N_i/N_j)^{f_q}$. As all our observables are intensive (i.e. densities), $f_q > 0$ indicates superextensive behaviour, $f_q = 0$ indicates extensive, and $f_q < 0$ subextensive behaviours. Table S3 gives the observed values of f_q.

D. Residuals of the best fit

To check whether systematic errors arise from our fitting procedure, Fig. S16 shows the residual errors—i.e. difference between the theoretical curves and the experimental data—for the five experiments shown in

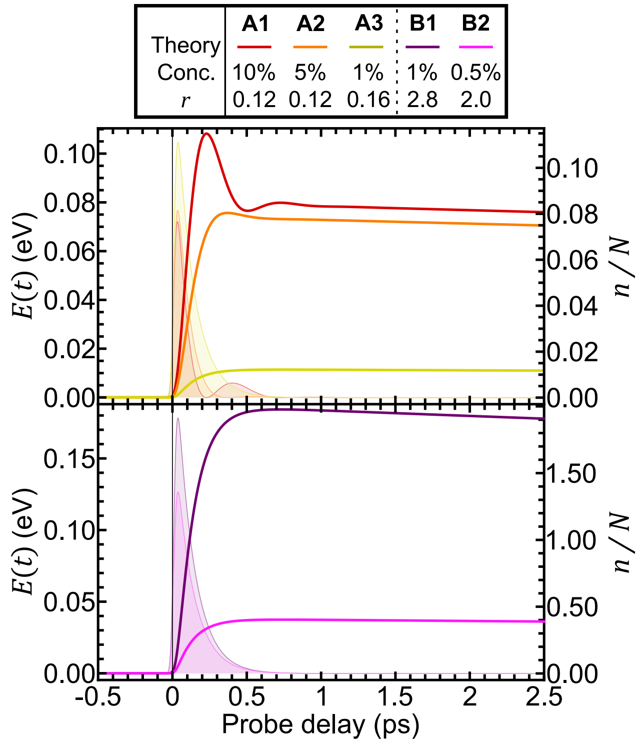

FIG. S15. **Further details of the dynamics of the quantum batteries**. These plots show the time dependence of energy without convolution by the instrument response function (lines without fill) and the ratio of the number of photons, n to molecules, N in the cavity (lines with fill).

Experiments	f_τ	$f_{E_{\max}}$	$f_{P_{\max}}$
**A1/A2**	-0.35	0.52	0.94
**A2/A3**	0.01	1.18	1.20
**B1/B2**	0.12	2.30	2.19

TABLE S3. **Observed subextensive and superextensive scaling behaviours in rise-time, stored energy, and charging power.** Power-law exponent f_q for observable $q \in \{\tau, E_{\max}, P_{\max}\}$, where $f_q > 0$, $f_q = 0$, $f_q < 0$ indicates superextensivity, extensivity, and subextensivity, respectively. Table values indicate that charging time τ is subextensive, whilst stored energy $E_{\max}$ and charging power $P_{\max}$ are superextensive. The first column indicates the corresponding experiments.

Figure 2 of the main text. As is clear, there are no discernible features in the residuals that are consistently present across the different experiments. This indicates that the theoretical curves account for the essential characteristics of the data.

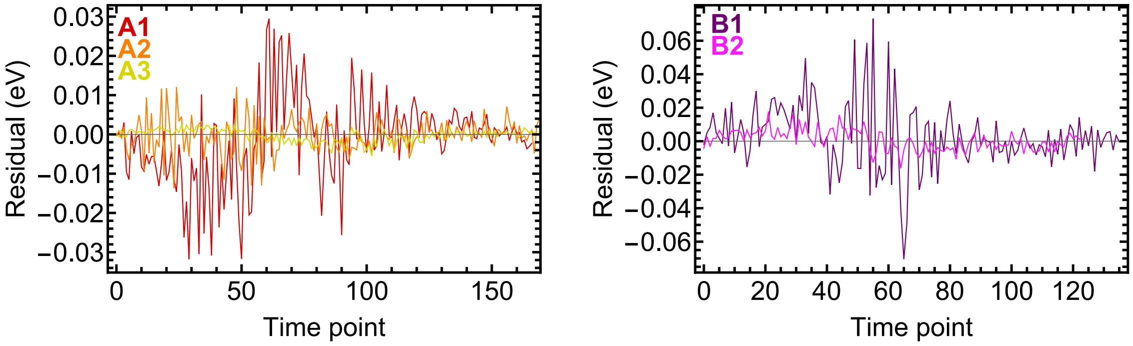

FIG. S16. The residuals of the theoretical curves and the experimental data over the duration of the experiments.

E. Theoretical fits for 185 fs lifetime

In Figure S17, we show the theoretical fits to the experimental data for experiments A1, A2, A3, B1 and B2 for a lifetime of 185 fs. This lifetime gave the optimal reduce chi-square in Figure S13. From the reduced chi-square fitting procedure, we found that the optimal parameters for this lifetime were $g = 16.9^{+1.7}_{-1.8}$ neV, $\gamma_0^z = 0.887^{+0.068}_{-0.060}$ meV and $\gamma^- = 0.0177^{+0.0033}_{-0.0029}$ meV.

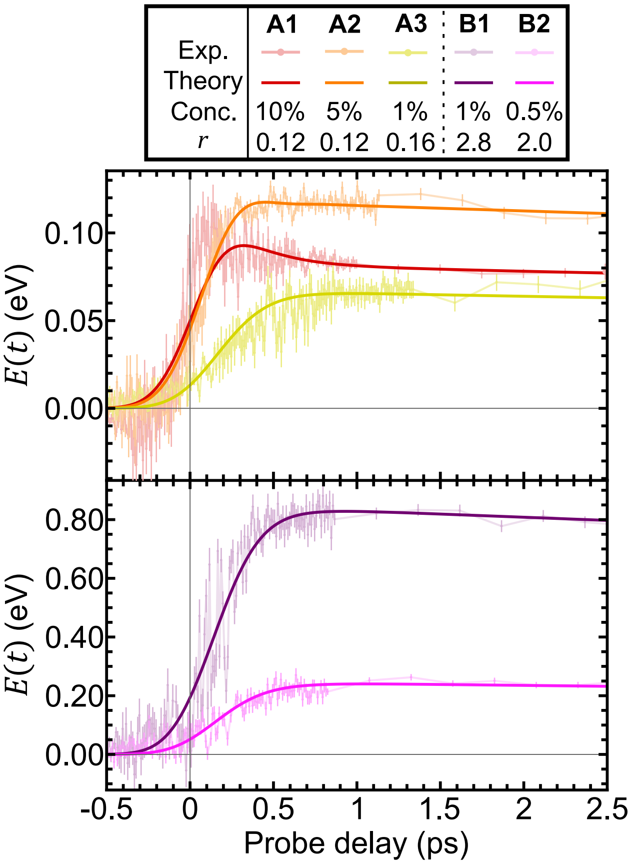

FIG. S17. The fits to the experimental data using a lifetime of 185 fs.

S4. MOVIE

Movie S1: Animation showing the molecular energy evolution for a range of N values.
Caption: The plots on the left-hand-side show the time evolution of the molecular energy over two different time scales. As the animation progresses, the value of N is increased, with the value indicated by the solid vertical line in the right-hand plot. Parameters are the same as those used in Figure S9.
File: Animation_LT120_Caption.mp4

REFERENCES AND NOTES

1. M. Gross, S. Haroche, Superradiance: An essay on the theory of collective spontaneous emission. *Phys. Rep.* **93**, 301–396 (1982).

2. N. Skribanowitz, I. P. Herman, J. C. MacGillivray, M. S. Feld, Observation of Dicke superradiance in optically pumped HF Gas. *Phys. Rev. Lett.* **30**, 309–312 (1973).

3. H. M. Gibbs, Q. H. F. Vrehen, H. M. J. Hikspoors, Single-pulse superfluorescence in cesium. *Phys. Rev. Lett.* **39**, 547–550 (1977).

4. J. Feldmann, G. Peter, E. O. Gobel, P. Dawson, K. Moore, C. Foxon, R. J. Elliott, Linewidth dependence of radiative exciton lifetimes in quantum-wells. *Phys. Rev. Lett.* **59**, 2337–2340 (1987).

5. B. Deveaud, F. Clerot, N. Roy, K. Satzke, B. Sermage, D. S. Katzer, Enhanced radiative recombination of free excitons in GaAs quantum wells. *Phys. Rev. Lett.* **67**, 2355–2358 (1991).

6. T. Itoh, M. Furumiya, Size-dependent homogeneous broadening of confined excitons in cucl microcrystals. *JOL* **48-49**, 704–708 (1991).

7. S. Deboer, D. A. Wiersma, Dephasing-induced damping of superradiant emission in J-aggregates. *Chem. Phys. Lett.* **165**, 45–53 (1990).

8. S. Inouye, A. P. Chikkatur, D. M. Stamper-Kurn, J. Stenger, D. E. Pritchard, W. Ketterle, Superradiant Rayleigh scattering from a Bose-Einstein condensate. *Science* **285**, 571–574 (1999).

9. R. Reimann, W. Alt, T. Kampschulte, T. Macha, L. Ratschbacher, N. Thau, S. Yoon, D. Meschede, Cavity-modified collective Rayleigh scattering of two atoms. *Phys. Rev. Lett.* **114**, 023601 (2015).

10. A. Angerer, K. Streltsov, T. Astner, S. Putz, H. Sumiya, S. Onoda, J. Isoya, W. J. Munro, K. Nemoto, J. Schmiedmayer, J. Majer, Superradiant emission from colour centres in diamond. *Nat. Phys.* **14**, 1168–1172 (2018).

11. K. D. Higgins, S. C. Benjamin, T. M. Stace, G. J. Milburn, B. W. Lovett, E. M. Gauger, Superabsorption of light via quantum engineering. *Nat. Commun.* **5**, 4705 (2014).

12. D. Yang, S.-h. Oh, J. Han, G. Son, J. Kim, J. Kim, M. Lee, K. An, Realization of superabsorption by time reversal of superradiance. *Nat. Photonics* **15**, 272–276 (2021).

13. R. Alicki, M. Fannes, Entanglement boost for extractable work from ensembles of quantum batteries. *Phys. Rev. E Stat. Nonlin. Soft Matter Phys.* **87**, 042123 (2013).

14. K. V. Hovhannisyan, M. Perarnau-Llobet, M. Huber, A. Acin, Entanglement generation is not necessary for optimal work extraction. *Phys. Rev. Lett.* **111**, 240401 (2013).

15. F. C. Binder, S. Vinjanampathy, K. Modi, J. Goold, Quantacell: Powerful charging of quantum batteries. *New J. Phys.* **17**, 075015 (2015).

16. G. M. Andolina, D. Farina, A. Mari, V. Pellegrini, V. Giovannetti, M. Polini, Charger-mediated energy transfer in exactly solvable models for quantum batteries. *Phys. Rev. B* **98**, 205423 (2018).

17. G. M. Andolina, M. Keck, A. Mari, M. Campisi, V. Giovannetti, M. Polini, Extractable work, the role of correlations, and asymptotic freedom in quantum batteries. *Phys. Rev. Lett.* **122**, 047702 (2019).

18. R. Alicki, A quantum open system model of molecular battery charged by excitons. *J. Chem. Phys.* **150**, 214110 (2019).

19. Y. Y. Zhang, T. R. Yang, L. Fu, X. Wang, Powerful harmonic charging in a quantum battery. *Phys. Rev. E* **99**, 052106 (2019).

20. F. Campaioli, F. A. Pollock, F. C. Binder, L. Celeri, J. Goold, S. Vinjanampathy, K. Modi, Enhancing the charging power of quantum batteries. *Phys. Rev. Lett.* **118**, 150601 (2017).

21. J. Q. Quach, W. J. Munro, Using dark states to charge and stabilize open quantum batteries. *Phys. Rev. Appl.* **14**, 024092 (2020).

22. T. P. Le, J. Levinsen, K. Modi, M. M. Parish, F. A. Pollock, Spin-chain model of a many-body quantum battery. *Phys. Rev. A* **97**, 022106 (2018).

23. X. Zhang, M. Blaauboer, Enhanced energy transfer in a Dicke quantum battery. arXiv:1812.10139 (2018).

24. D. Ferraro, M. Campisi, G. M. Andolina, V. Pellegrini, M. Polini, High-power collective charging of a solid-state quantum battery. *Phys. Rev. Lett.* **120**, 117702 (2018).

25. S. Gherardini, F. Campaioli, F. Caruso, F. C. Binder, Stabilizing open quantum batteries by sequential measurements. *Phys. Rev. Res.* **2**, 013095 (2020).

26. A. C. Santos, A. Saguia, M. S. Sarandy, Stable and charge-switchable quantum batteries. *Phys. Rev. E* **101**, 062114 (2020).

27. W. M. Brown, E. M. Gauger, Light harvesting with guide-slide superabsorbing condensed-matter nanostructures. *J. Phys. Chem. Lett.* **10**, 4323–4329 (2019).

28. D. Sanvitto, S. Kéna-Cohen, The road towards polaritonic devices. *Nat. Mater.* **15**, 1061–1073 (2016).

29. V. Savona, L. C. Andreani, P. Schwendimann, A. Quattropani, Quantum well excitons in semiconductor microcavities: Unified treatment of weak and strong coupling regimes. *Solid State Commun.* **93**, 733–739 (1995).

30. G. Cerullo, C. Manzoni, L. Lüer, D. Polli, Time-resolved methods in biophysics. 4. Broadband pump–probe spectroscopy system with sub-20 fs temporal resolution for the study of energy transfer processes in photosynthesis. *Photochem. Photobiol. Sci.* **6**, 135–144 (2007).

31. C. Manzoni, G. Cerullo, Design criteria for ultrafast optical parametric amplifiers. *J. Opt.* **18**, 103501 (2016).

32. O. Svelto, D. C. Hanna, *Principles of Lasers* (Springer, 2010), vol. 1.

33. T. Virgili, D. G. Lidzey, D. D. C. Bradley, G. Cerullo, S. Stagira, S. De Silvestri, An ultrafast spectroscopy study of stimulated emission in poly(9,9-dioctylfluorene) films and microcavities. *Appl. Phys. Lett.* **74**, 2767–2769 (1999).

34. P. Kirton, J. Keeling, Suppressing and restoring the Dicke superradiance transition by dephasing and decay. *Phys. Rev. Lett.* **118**, 123602 (2017).

35. K. B. Arnardottir, A. J. Moilanen, A. Strashko, P. Törmä, J. Keeling, Multimode organic polariton lasing. arXiv:2004.06679 (2020).

36. M. Zens, D. O. Krimer, S. Rotter, Critical phenomena and nonlinear dynamics in a spin ensemble strongly coupled to a cavity. II. Semiclassical-to-quantum boundary. *Phys. Rev. A* **100**, 013856 (2019).

37. K. D. B. Higgins, B. W. Lovett, E. M. Gauger, Quantum-enhanced capture of photons using optical ratchet states. *J. Phys. Chem. C* **121**, 20714–20719 (2017).

38. C. P. Dietrich, A. Steude, L. Tropf, M. Schubert, N. M. Kronenberg, K. Ostermann, S. Hofling, M. C. Gather, An exciton-polariton laser based on biologically produced fluorescent protein. *Sci. Adv.* **2**, e1600666 (2016).

39. Y. Wang, P. Shen, J. Liu, Y. Xue, Y. Wang, M. Yao, L. Shen, Recent advances of organic solar cells with optical microcavities. *Solar RRL* **3**, 1900181 (2019).

40. B. Kippelen, J.-L. Brédas, Organic photovoltaics. *Energ. Environ. Sci.* **2**, 251–261 (2009).

41. K. A. Mazzio, C. K. Luscombe, The future of organic photovoltaics. *Chem. Soc. Rev.* **44**, 78–90 (2015).

42. G. J. Hedley, A. Ruseckas, I. D. W. Samuel, Light harvesting for organic photovoltaics. *Chem. Rev.* **117**, 796–837 (2017).

43. P. Cheng, G. Li, X. Zhan, Y. Yang, Next-generation organic photovoltaics based on non-fullerene acceptors. *Nat. Photonics* **12**, 131–142 (2018).

44. E. Hecht, *Optics* (Pearson Education Incorporated, 2017).

45. D. Wang, H. Kelkar, D. Martin-Cano, D. Rattenbacher, A. Shkarin, T. Utikal, S. Götzinger, V. Sandoghdar, Turning a molecule into a coherent two-level quantum system. *Nat. Phys.* **15**, 483–489 (2019).

46. C. Gardiner, *Handbook of Stochastic Methods for Physics, Chemistry, and the Natural Sciences Springer* (ed. 4, 2009).

47. J. del Pino, J. Feist, F. J. Garcia-Vidal, Quantum theory of collective strong coupling of molecular vibrations with a microcavity mode. *New J. Phys.* **17**, 053040 (2015).

48. J. V. Wall, C. R. Jenkins, *Practical Statistics for Astronomers* (Cambridge Univ. Press, 2003).

49. L. V. Wang, H.-i. Wu, *Biomedical Optics: Principles and Imaging* (John Wiley & Sons, 2012).

50. K. Yamashita, U. Huynh, J. Richter, L. Eyre, F. Deschler, A. Rao, K. Goto, T. Nishimura, T. Yamao, S. Hotta, H. Yanagi, M. Nakayama, R. H. Friend, Ultrafast dynamics of polariton cooling and renormalization in an organic single-crystal microcavity under nonresonant pumping. *ACS Photonics* **5**, 2182–2188 (2018).
